# ZINBA integrates local covariates with DNA-seq data to identify broad and narrow regions of enrichment, even within amplified genomic regions

**DOI:** 10.1186/gb-2011-12-7-r67

**Published:** 2011-07-25

**Authors:** Naim U Rashid, Paul G Giresi, Joseph G Ibrahim, Wei Sun, Jason D Lieb

**Affiliations:** 1 Department of Biostatistics, Gillings School of Global Public Health, The University of North Carolina at Chapel Hill, Chapel Hill, NC 27599, USA; 2 Department of Biology, Carolina Center for Genome Sciences, and Lineberger Comprehensive Cancer Center, The University of North Carolina at Chapel Hill, Chapel Hill, NC 27599, USA; 3 Department of Genetics and School of Medicine, University of North Carolina at Chapel Hill, Chapel Hill, NC 27599, USA

## Abstract

ZINBA (Zero-Inflated Negative Binomial Algorithm) identifies genomic regions enriched in a variety of ChIP-seq and related next-generation sequencing experiments (DNA-seq), calling both broad and narrow modes of enrichment across a range of signal-to-noise ratios. ZINBA models and accounts for factors that co-vary with background or experimental signal, such as G/C content, and identifies enrichment in genomes with complex local copy number variations. ZINBA provides a single unified framework for analyzing DNA-seq experiments in challenging genomic contexts.

Software website: http://code.google.com/p/zinba/

## Background

Next generation sequencing (NGS) technologies are now routinely utilized for genome-wide detection of DNA fragments isolated by a diverse set of assays interrogating genomic processes [1]. We refer to these collectively as DNA-seq experiments, which include chromatin immunoprecipitation (ChIP-seq), DNase hypersensitive site mapping (DNase-seq) [2], and formaldehyde-assisted isolation of regulatory elements (FAIRE-seq) [3], among others. Several algorithms are currently available for the identification of genomic regions enriched by a given experiment. Although each is well suited for the analysis of a particular intended data type, the underlying assumptions are not always suitable for the multitude of possible enrichment patterns found in DNA-seq datasets [4]. An algorithm capable of robust detection of enrichment across a multitude of enrichment patterns, with performance comparable to the existing set of algorithms specific to each data type, would have high utility.

**Figure 1:**
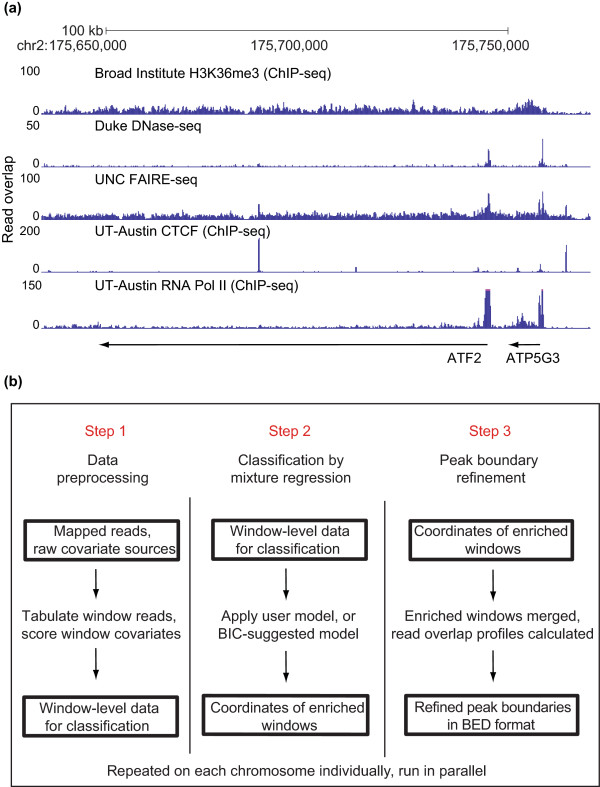
**ZINBA provides a unified framework for the detection of enriched sites across a wide variety of DNA-seq datasets**. **(a) **A 100-kb region of chromosome 2 at the *ATF2 *gene locus illustrating the diversity of enrichment patterns in DNA-seq data, which includes histone H3 lysine 36 tri-methylation (H3K36me3), CCCTC-binding factor (CTCF) and RNA polymerase II (RNA Pol II) ChIP-seq along with the FAIRE-seq and DNase-seq assays. Data for each of the DNA-seq experiments are displayed as the number of overlapping extended reads at each base pair, which was produced by the indicated groups and is available from the UCSC genome browser. **(b) **ZINBA comprises three steps that can each operate as an independent module. In step 1, the set of aligned reads from the experiment along with a set of covariate measures are collated for each contiguous non-overlapping window spanning the genome. In step 2, the component-specific model formulations of covariates are employed by the mixture regression framework to compute the posterior probability of each window belonging to either the zero-inflated, background or enriched components. The component-specific model formulations of covariates can be generated using an automated model selection procedure or specified by the user. In step 3, the windows exceeding the user-specified probability threshold (default 0.95) are merged to form broad regions of enrichment and a shape detection algorithm is employed on the read overlap representation of the data to refine the boundary estimates of distinct punctate peaks. BED, browser extensible data; BIC, Bayesian information criterion.

For example, regions of ChIP-seq enrichment for transcription factors [5-16] typically comprise a small proportion of the genome (< 1%), are short (< 500 bp), and have relatively high signal-to-noise ratios. Histone modification data [2,6] can vary widely in terms of length of enriched regions (Figure 1a), the proportion of the genome enriched [4], and the signal-to-noise ratio. To assess the statistical significance of an identified enriched region, assumptions regarding the distribution of signal in background and enriched regions must be made. The majority of algorithms perform optimally for the identification of transcription factor binding sites (TFBSs) from ChIP-seq data [17]. However, as the proportion of the genome that is enriched increases and/or the signal-to-noise ratio decreases compared with TFBS data [2,6,18-20] the performance of many existing tools declines [17,19,21-23]. Researchers interested in the analysis of several types of data for a given experiment must often combine results from different algorithms. In addition, NGS data often contain biases due to several factors, including G/C content [24-26] and mappability [6]. Data from a matched input control sample may control for the effects of such confounding factors [27], but input data are often not available, and it is unclear whether input alone is sufficient to model background signals in DNA-seq data.

To address these issues, we introduce a flexible statistical framework called ZINBA (Zero-Inflated Negative Binomial Algorithm) that identifies genomic regions enriched for sequenced reads across a wide spectrum of signal patterns and experimental conditions. ZINBA implements a mixture regression approach, which probabilistically classifies genomic regions into three general components: background, enrichment, and an artificial zero count. The regression framework allows each of the components to be modeled separately using a set of covariates, which leads to better characterization of each component and subsequent classification outcomes. In addition, the mixture-modeling approach affords ZINBA the flexibility to determine the set of genomic regions comprising background without relying on any prior assumptions of the proportion of the genome that is enriched. Following classification, neighboring regions classified as enriched are merged and boundaries of punctate signal within enriched regions are determined, allowing the isolation of both broad and narrow elements.

We applied ZINBA to FAIRE-seq and ChIP-seq of CCCTC-binding factor (CTCF), RNA polymerase II (RNA Pol II), and histone H3 lysine 36 tri-methylation (H3K36me3) (Figure 1a). These datasets represent a diversity of signal patterns ranging from narrow peaks with high signal-to-noise ratios (CTCF) to broad enrichment regions with low signal-to-noise ratios (H3K36me3). In addition to identifying biologically relevant signals in each of these datasets, ZINBA is capable of estimating the contribution of component-specific covariates to signal in each component. Incorporation of covariates into the model improved peak detection in difficult modeling situations, such as in amplified genomic regions. In the absence of input control, we show that other covariates allow for comparable performance as when input control is utilized. Lastly, we demonstrate that ZINBA's ability to isolate broad and narrow enrichment regions reveals functional differences in RNA Pol II elongation status. We conclude that ZINBA provides a general and flexible framework for the analysis of a diverse set of DNA-seq datasets.

## Results

### ZINBA overview

ZINBA performs three steps: data preprocessing, determination of significantly enriched regions, and an optional boundary refinement for more narrow sites (Figure 1b). The first step involves tabulating the number of reads falling into contiguous non-overlapping windows (default 250 bp) tiled across each chromosome and scoring corresponding covariate information. Covariates can consist of any quantity that may co-vary with signal in a given region, including, for example, G/C content, a smoothed average of local background, read counts for an input control sample, or the proportion of mappable [28] bases, which we define as the mappability score (Materials and methods). Optionally, additional sets of contiguous windows with offset starting positions can be tabulated for increased resolution. Each set of offset windows is analyzed independently in the next step.

In the second step, a novel mixture regression model is used to probabilistically classify each window into one of three components: background, enrichment, or zero-inflated. In this context, and throughout the manuscript, the term 'enrichment' will refer to genomic DNA sequences that were captured specifically as the result of the biological experiment under consideration. The term 'background' includes genomic DNA sequences that appear due to experimental noise, noise that arises in the sequencing process, or noise that arises in the computational processing of the data. The term 'zero-inflated' refers to those genomic locations at which we might expect coverage by a sequencing read derived from either the background or enrichment signal components, but that are not represented in the real data. Zero-inflation typically occurs due to a lack of sequencing depth and is common in many NGS datasets. Regions containing higher proportions of non-mappable bases are also more likely to be zero-inflated, as it is more difficult to assign reads to these regions during the mapping process.

ZINBA utilizes an iterative approach [29] to determine for each window the relative likelihood of belonging to each component, in addition to estimating the relationship between average signal in each component and a set of covariates (Materials and methods). Each iteration consists of two steps. In the first step, a set of posterior probabilities of component membership is computed for each window, based on how well each window fits with the average signal level in each component, adjusted for covariate effects. In the next step, the average signal level in each component is modeled separately with its own formulation of covariates using weighted generalized linear models (GLMs). The posterior probabilities of component membership are used as regression weights and serve to partition the genome into likely background, enrichment, and zero-inflated regions to determine component signal. The model iterates between these two steps until the classification and component-specific covariate estimates cease to change.

Adjusting for covariate effects is often beneficial or necessary for dissecting enrichment regions and background. For example, although signal in background regions is typically lower than in regions of enrichment, background regions in copy-number amplified regions may have higher signal than enrichment regions that occur in locations with a normal DNA copy number. Thus, adjusting for copy number changes is necessary for correct separation of background and enrichment regions. The set of covariates used to model each component can be selected based on either prior knowledge or an information criterion, such as the Bayesian information criterion (BIC). Covariates with no or weak relationships with mean signal in a component will have little effect on classification, but do contribute to model complexity. The BIC criterion helps to remove such covariates to balance model fit and model size.

In the third step, all overlapping or adjacent windows classified as enriched are merged. For the detection of broader elements, especially helpful for histone modifications demarcating broad genomic regions (such as H3K36me3), an additional 'broad' setting is available that merges enriched windows within a fixed distance. An optional shape-detection algorithm may then be applied to identify sharp enrichment signals within broader enriched regions.

### Modeling signal components with relevant covariates improves enrichment detection

To evaluate the utility of incorporating covariate information for the detection of enriched regions, we constructed simulated datasets, and used G/C content as one example of such a covariate. Simulated datasets were constructed to artificially control the relationship between G/C content and the enrichment, background, and zero-inflated components. Window count data were simulated to represent three types of common NGS signal patterns, ranging from TFBSs (high signal-to-noise ratio, 1% of genome belongs to enrichment component), FAIRE (moderate signal-to-noise ratio, 5% of genome belongs to enrichment component), to some histone modifications (low signal-to-noise ratio, 10% of genome belongs to enrichment component). For each data type, three sets of data were simulated, hence nine datasets in total. In each data set, G/C content always had a positive relationship with signal in the background component and a positive relationship with the probability of being zero-inflated. However, G/C content was simulated to have either a positive, neutral or negative relationship with enrichment. For each of the nine datasets, 100,000 windows were simulated. These consisted of 250-bp windows from human chromosome 22 (Materials and methods). G/C content was simulated from these windows as well.

Now, for each of the nine simulated datasets, three different uses of the covariate were employed to model the simulated data: (a) model 1, no covariates; (b) model 2, G/C content is incorporated in modeling the zero-inflated and background components only; (c) model 3, G/C content is incorporated in modeling all three components.

**Figure 2:**
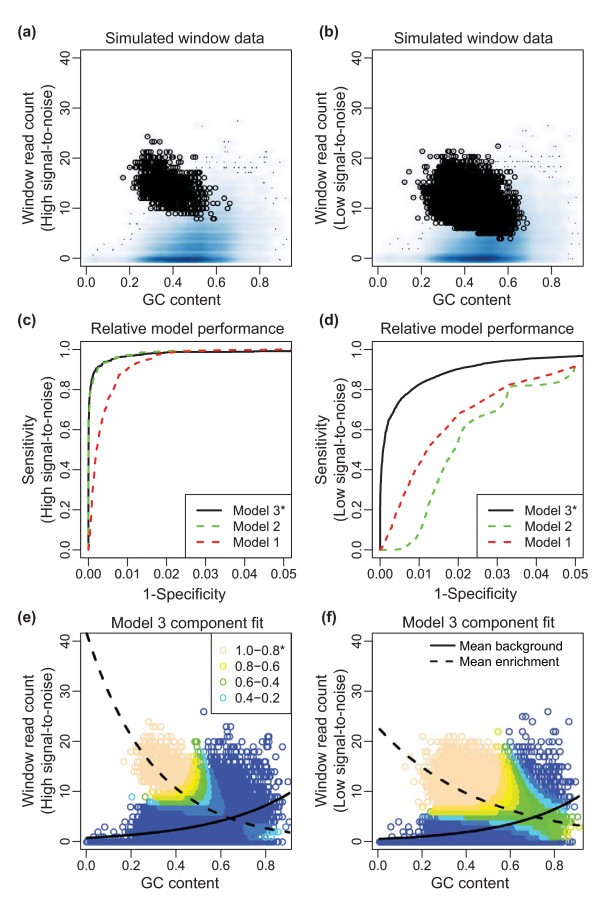
**Accounting for relevant component-specific covariates results in the optimal classification of background and enriched components for a simulated data set**. **(a, b) **Density plots showing the distribution of background (blue shading) and enriched (black circles) simulated counts (y-axis) versus G/C content (x-axis). Window counts were simulated with either (a) a low proportion of high signal-to-noise sites or (b) a high proportion of low signal-to-noise sites. In this example G/C content had a positive and negative relationship with the background and enriched components, respectively. **(c, d) **Receiver operating characteristic (ROC) curves for the performance of three different component-specific covariate model formulations, including no covariates (model 1, red dashed line), G/C content modeling the background and zero-inflated components (model 2, green dashed line) and G/C content modeling the background, zero-inflated and enriched components (model 3, black solid line). Classification results for the simulated (c) low proportion of high signal-to-noise sites and (d) high proportion of low signal-to-noise sites. Utilization of relevant covariates in each component resulted in better classification outcomes (model 3). This impact is greater in lower signal-to-noise data (d), where it is more difficult to distinguish enrichment from background. **(e, f) **Scatter plot of G/C content (x-axis) versus simulated window counts (y-axis) using model 3 to estimate the posterior probability of a window being enriched, which is depicted as a color gradient. Lighter colors correspond to higher posterior probability and a greater likelihood of being enriched. Posterior probabilities for the simulated (e) low proportion of high signal-to-noise sites and (f) high proportion of low signal-to-noise sites are shown along with model estimates for the background (solid black line) and enriched components (dashed black line).

Our results show that models that properly accounted for the underlying simulated relationships with G/C content in each component resulted in the best classification outcomes. For example, when enrichment had an inverse relationship with G/C content (Figure 2a, b), model 3 consistently led to higher sensitivity and specificity relative to models 1 and 2 (Figure 2c, d). Simulated component-specific relationships between G/C content and signal were also correctly captured in model 3 (Figure 2e, f), with average enrichment signal decreasing and average background signal increasing with respect to G/C content. Ignoring the role of G/C content completely (model 1) resulted in classification based purely on signal, which misses informative trends in the data (Figure S1 in Additional file 1). We find similar results for the simulated condition of positive and neutral relationships between G/C content and enrichment (Figures S2 and S3 in Additional file 1). Thus, including relevant covariates to model each component provides a more informed assessment of enrichment versus background.

These results also serve to illuminate how ZINBA distinguishes the separate roles of component-specific covariates. For example, covariates that are relevant to the background component explain variability in background signal that may otherwise be confused for enrichment. This benefit of ZINBA is more apparent when the signal-to-noise ratio is low (Figure 2b, d, f) because, in that case, many background and enrichment windows contain similar numbers of reads, and the two states are difficult to distinguish by signal alone. In the situation where we simulated a neutral relationship of G/C content with enrichment, model 3 had similar performance to model 2, suggesting that the use of G/C content to model the enrichment component did not degrade classification performance. Rather, the estimated effect of G/C content in the enrichment component was close to zero, and thus had little effect on classification (Figure S2 in Additional file 1) at the cost of greater model complexity.

While we chose to simulate our data in this section with respect to only one covariate, the regression basis for the mixture model allows the inclusion of multiple covariates simultaneously, as is inherent in any regression-based framework. Regardless of whether the data consist of rare, high signal-to-noise enrichment or common, low signal-to-noise enrichment, the model performs better when each component is modeled with relevant sets of covariates. However, the performance gain when using relevant covariates is greatest in lower signal-to-noise data.

### Automated model selection

Relevant covariates are not always known *a priori*. To discover the appropriate formulation of covariates for each component, ZINBA employs the BIC [30] to select the best model among all possible models, given a set of starting covariates (Materials and methods). BIC balances model fit and model complexity and has long been employed as a statistical assessment of model performance. The regression framework inherent in ZINBA also allows for the modeling of interactions between covariates. Therefore, all pair-wise and three-way interactions between the starting covariates for each component are considered in the model selection procedure. The automated model selection procedure was able to select the most appropriate model for all nine simulated conditions from the previous section.

### ZINBA detects relationships between covariates and component signal that vary by experiment

Evaluation of the relationships between the set of component-specific covariates selected using the automated model selection procedure and the datasets shown in Figure 1a[31,32] revealed that our mappability score and input control were positively related with mean background signal in each ChIP-seq dataset, which is consistent with previous reports [5,28]. Each dataset exhibits distinctly different degrees of signal-to-noise ratio, length of enriched regions, and total proportion of the genome enriched. These differences can be attributed to both functional differences related to biological activity and technical aspects of the different assays. However, the relationship between G/C content and background signal was not consistent between different DNA-seq experiments (Table S1 in Additional file 1), nor were they consistent between components of the same dataset.

**Figure 3:**
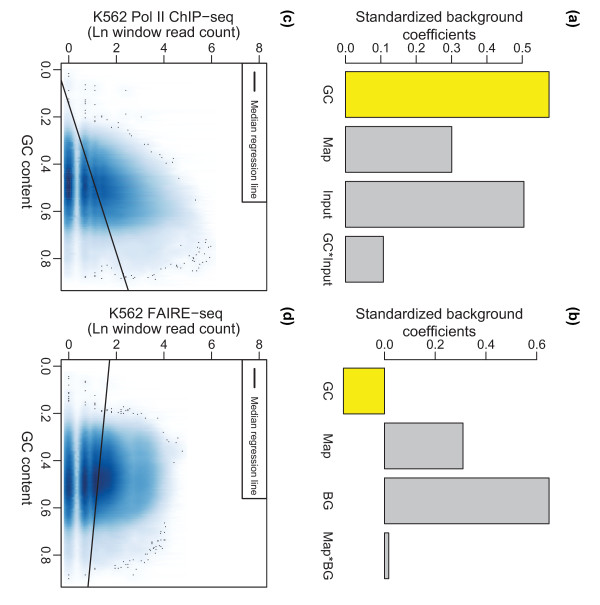
**Estimates of covariate effects differ among DNA-seq data types**. **(a, b) **Estimates for the set of BIC selected covariates for the background components of the (a) RNA Pol II ChIP-seq and (b) FAIRE-seq data from chromosome 22 in K562 cells. The set of covariates was standardized to a mean of 0 and variance of 1, which included G/C content ('GC'), mappability score ('Map'), the local background estimate ('BG'), and input control ('Input'). The G/C content covariate (yellow bars) had an opposing effect on the background component for the RNA Pol II (positive) (a) and FAIRE (negative) (b) data. **(c, d) **Density plots of G/C content (x-axis) versus the natural log of window read count (y-axis) in non-enriched windows (enrichment posterior probability < 0.50) from the (c) RNA Pol II and (d) FAIRE data. Median regression lines fit to the set of background windows from each dataset parallel the ZINBA-estimated relationships between G/C content and signal in background regions.

For the RNA Pol II and CTCF data, model estimates reveal that G/C content had a positive relationship in background regions, similar to previous reports on G/C content bias [24-26] (Figure 3a). However, in FAIRE-seq data, G/C content was negatively associated with the background component (Figure 3b). These differences can easily be observed from scatter plots of the raw read counts from windows classified as background versus the corresponding G/C content for the RNA Pol II ChIP-seq and FAIRE-seq datasets (Figure 3c, d). The exact cause of the differences in the relationship between G/C content and background signal between datasets, and whether it could be technical or biological, is not known.

The relationship for each covariate also differed in magnitude and direction across components of the same dataset. For example, in FAIRE-seq data, while there was a negative relationship with G/C content in background regions, there was a positive relationship in enriched regions (Table S1 in Additional file 1). A similar difference between the relationship of G/C content in the background and enrichment regions was found for the RNA Pol II ChIP-seq data. Thus, the relationships of covariates with background signal may not be consistent across different data types, and may differ in their relationships to signal in background and enrichment regions of the same data type.

An input control may be used to account for the relationships of G/C content and mappability with background signal. However, the model estimates suggest that input data alone may not explain all of the variability in DNA-seq background. Examination of the relationships of covariates with input signal and DNA-seq background reveals differences in the effects of covariates within each (Figure S4 in Additional file 1). In the case of RNA Pol II (Figure S4a, b in Additional file 1) and CTCF (Figure S4c, d in Additional file 1), where the estimated relationship of G/C content with background DNA-seq signal is positive, in the matching input control sample the relationship with G/C content is relatively neutral. The reason for these differences is currently unknown, but may be related to sample handling differences between the ChIP and input samples.

### Incorporation of a covariate for copy number allows peak calling within amplified genomic regions

**Figure 4:**
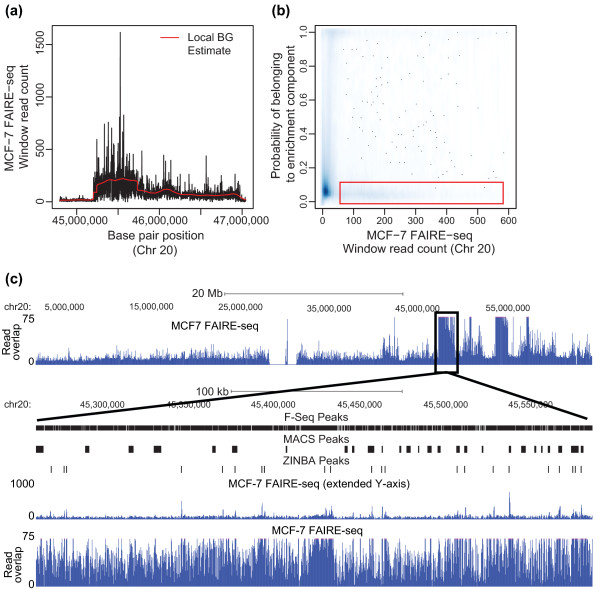
**Covariate-mediated adjustment of classification aids in the discrimination of background and enriched regions**. **(a) **The local background (BG) estimate (red line) approximates a CNV detected by FAIRE-seq (black line) within a 2-Mbp region of chromosome 20 in MCF-7 cells. **(b) **Density plot of the window read counts for FAIRE-seq data in MCF-7 (chromosome 20) versus the posterior probability of a given window being classified as enriched, which included the local background estimate as a covariate in the ZINBA model formulation. The red box highlights a set of windows with high read counts (CNV background) being assigned a low posterior probability of being enriched. **(c) **The read overlap representation of MCF-7 FAIRE-seq data for all of chromosome 20 (top row) is displayed in the UCSC Genome Browser. The bottom panels zoom in on the black box outlining a CNV (same as panel (a)). Here a set of peak calls by F-Seq, MACS and ZINBA are shown as black boxes along with the FAIRE-seq data displayed using either an extended (top) or standard y-axis.

One challenge for the analysis of DNA-seq data is fluctuations in background signal resulting from copy number variations (CNVs). If not properly accounted for, such changes in background can result in significant false positives. This is especially true if there are no input control samples for comparison, or if the input control samples are insufficiently sequenced. To account for this, we constructed a new covariate to measure local background, and included this covariate in our mixture regression framework to account for local copy number changes. Changes in background signal levels due to CNVs were estimated locally using the DNA-seq sample itself, supplemented by a change-point detection method to determine boundaries of likely CNVs (Materials and methods). Application of this approach provided an accurate estimation of signal changes due to local CNVs in a FAIRE-seq MCF-7 dataset, which is aneuploid and has extensive CNVs [33] (Figure 4a).

Using a BIC-selected model considering the local background estimate, G/C content, and mappability score as starting covariates, we found ZINBA was able to correctly classify background regions within CNVs (Figure 4b) and called 8 and 11 times fewer peaks (1,258) using a FAIRE-seq dataset in MCF-7 CNV regions in chromosome 20 [34] relative to MACS [5] and F-seq [35] (Figure 4c). Incorporation of this covariate also leads to the better recovery of relevant peak regions within ENCODE [36] datasets, as we demonstrate in later sections.

Estimation of local background from the experimental data is only effective when local background is sampled from a sufficiently large window size, where these large windows (default 100 kb) will not be dominated by enriched signal. This is the case with the majority of data types, as most contain enriched features that span no more than several kilobases. In any case, the flexibility of ZINBA allows for CNV estimates from any source to be included into the model selection procedure and determination of enrichment. ZINBA also includes a 'CNV mode', which can be run on input DNA for a quick estimation of the extent of amplified genomic regions in a given sample. This mode utilizes 10-kb windows in the ZINBA mixture model without any covariates, aiming to detect extended region enrichment of input reads.

### Evaluation of ZINBA over a wide range of signal patterns and amplitudes

We selected a variety of DNA-seq datasets, including FAIRE-seq, CTCF, RNA Pol II, and H3K36me3 ChIP-seq, to compare the performance of ZINBA with other existing methods across a range of signal-to-noise ratios, patterns of enrichment, and proportion of total genomic enrichment. For example, CTCF ChIP-seq data exhibit punctate, high signal-to-noise ratio peaks, FAIRE-seq data have broader, low signal-to-noise ratio peaks, and RNA Pol II ChIP-seq data contain a mixture of punctate high signal-to-noise and diffuse low signal-to-noise peaks. H3K36me3 enrichment encompasses very broad domains of many kilobases, extending over large portions of transcribed regions. For each dataset, we applied the automated model selection tool to determine the set of component-specific covariates to model each dataset (Materials and methods).

ZINBA was compared with MACS [5] and F-Seq [2], which represent two classes of peak calling algorithms that also do not require an input control sample to call regions of enrichment. MACS [5] represents a class of algorithms that uses a sliding window approach for the detection of enriched regions compared to a matching input control sample or local background estimate. F-Seq [17] represents a class of algorithms that use kernel density estimation to estimate local read density and identifies enriched regions as those with a kernel density estimation larger than a user-defined threshold, which is estimated using simulations assuming random assortment of sample reads.

For each algorithm, the top N set of ranked peaks (500, 1,000, 2,000, and so on) were selected. The performance of each was evaluated by calculating the average peak length, the proportion of peaks overlapping a set of biologically significant features (within 150 bp) and the average distance to these features. For ZINBA, the set of unrefined peak calls (merged enriched windows) and refined peak calls (boundaries of punctate peaks within merged regions) were evaluated separately to determine their relative utility in each dataset. For the H3K36me3 data, we utilized the ZINBA 'broad' setting (Materials and methods) to capture regions of enrichment that may extend for many kilobases.

#### All algorithms perform comparably for the analysis of punctate high signal-to-noise datasets

**Figure 5:**
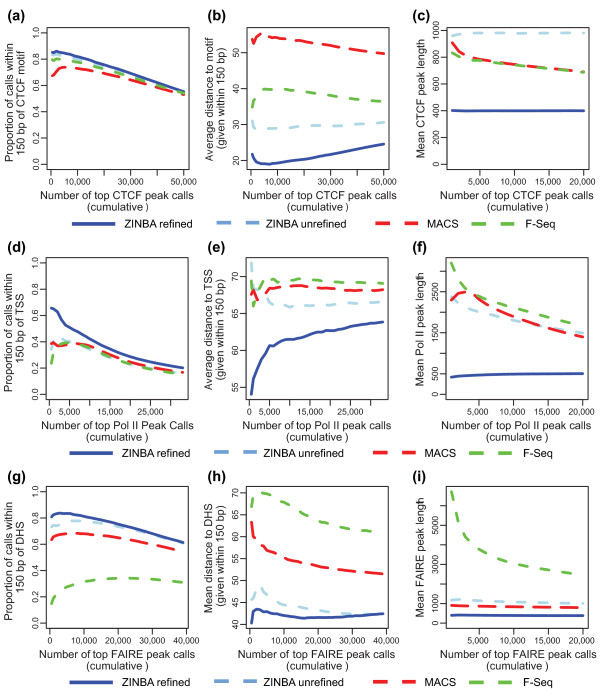
**Robust detection of biologically relevant features across a variety of DNA-seq data types by ZINBA**. **(a-i) **For CTCF ChIP-seq (a-c), RNA Pol II ChIP-seq (d-f) and FAIRE-seq (g-i) data, the top N ranked peaks from MACS (red dashed line), F-Seq (green dashed line) and ZINBA unrefined regions (light blue dashed line), and ZINBA refined regions (blue solid line) were compared based on the proportion overlapping a biologically relevant set of features (a, d, g), average distance to the biologically relevant set of features (b, e, h) and average length of peaks (c, f, i). The biologically relevant set of features included the CTCF motif (a), transcription start sites (TSSs) for RNA Pol II (d) and DNase hypersensitive sites (DHSs) for FAIRE (g).

For the CTCF ChIP-seq data set, the set of ranked peaks for each algorithm was compared to the occurrence of the CTCF motif (JASPAR motif MA0139.1). The genome-wide set of motifs was identified using FIMO, part of the MEME suite [37], with default parameters. All of the algorithms were able to identify a high proportion of sites containing the CTCF motif (Figure 5a) and had comparable peak lengths (Figure 5c). Positioning of peaks called by ZINBA was slightly closer to the CTCF motifs (Figure 5b). These results are consistent with other comparisons of ChIP-seq peak calling algorithms [17], which revealed few differences in sensitivity and specificity when applied to high signal-to-noise ChIP-seq data. Of the 50,228 refined peaks called by ZINBA, 95.2% were in common with MACS (60,135 peaks) and 99.9% were in common with F-seq (276,879 peaks).

#### The set of broad and punctate peaks identified by ZINBA for RNA Pol II ChIP-seq data reflects the elongation status of the polymerase

One unique feature of RNA Pol II ChIP-seq data is that enrichment consists of both punctate high signal-to-noise ratio peaks at transcription start sites (TSSs) and broader, low signal-to-noise peaks into the body of genes [4]. All of the algorithms were able to capture a large proportion of annotated TSSs (Figure 5d, e; Figure S5a in Additional file 1). However, the set of refined peaks called by the shape detection algorithm within ZINBA resulted in a set of narrower peaks much more closely associated with the TSSs of genes (Figure 5e, f) compared with MACS, F-Seq, and unrefined ZINBA peak calls. A relatively high degree of overlap can be seen between each of the peak sets, although the overlap is not as strong compared to those observed for the CTCF dataset (Figure S5b in Additional file 1).

The ability to produce both a refined (punctate) and unrefined (broad) set of peak calls using ZINBA provides an opportunity to infer elongating versus stalled RNA Pol II. For the case of stalled RNA Pol II, one would expect a punctate peak at the TSS, but no broad peak within the body of the gene [38]. Under this expectation, we computed a 'stalling score' (Materials and methods), where smaller values correspond to a broad high-amplitude signal across the gene, and larger values to a punctate signal near the 5' end of the gene and lower-amplitude signal along the gene body. Previous computations of RNA Pol II stalling scores utilized a height ratio between the punctate peak at the TSS and the median height of the broader region [39] (Figure S6a in Additional file 1). Using ZINBA, our stalling score further incorporates the lengths of the broad and punctate enriched regions found in the experimental sample. The stalling index had a strong negative relationship (*P*-value < 10^-10^) to the expression of the nearby gene (Figure S6b in Additional file 1) and explained more of the variance in measured gene expression (R^2 ^= 3.5%) than a score utilizing only the ratio of punctate to broad signal height (R^2 ^= 0.04%). The ability to calculate this metric reflects one potential use of the peak boundary refinement module within the ZINBA framework.

#### ZINBA accurately identifies regions of enrichment in low signal-to-noise datasets without the use of input for background estimation

FAIRE-seq [3,40] differs from ChIP-seq in that it is an antibody-free method that recovers DNA fragments that are relatively resistant to formaldehyde crosslinking to proteins. The crosslinking profile of chromatin is likely dominated by histone-DNA interactions, and therefore the sites preferentially recovered by FAIRE correspond to sites of nucleosome depletion. On average the size of each FAIRE site corresponds to the loss of approximately one nucleosome (200 to 300 bp). Compared to the binding events identified for TFBSs by ChIP-seq, the FAIRE-seq sites tend to have much lower signal-to-noise, have a slightly broader pattern of enrichment, and encompass a larger proportion (1 to 2%) of the genome. In addition, input control is often not available. Therefore, many of the assumptions utilized by existing algorithms, especially for the analysis of TFBS ChIP-seq, are not well-suited to the analysis of this data type [22].

We analyzed a K562 FAIRE-seq dataset lacking a matching input control sample with each algorithm, and compared the resulting set of peaks from each algorithm to a set of DNase I hypersensitivity sites (DHSs) [31,32] isolated from the exact same set of cells. The DHSs were called by F-seq, and were selected as a standard because of the longstanding use of DNase as a method for identification of open chromatin sites. Both ZINBA and MACS called a high proportion of FAIRE sites that overlapped a DHS, but a low proportion of FAIRE sites called by F-seq were localized to a DHS (Figure 5g). The set of sites called by both MACS and F-Seq tended to be longer and more errant in K562 CNV regions [31,32] (Figure S7a in Additional file 1), where approximately 37% of MACS and 27% of F-seq peaks were localized to a DHS, compared to 50% of ZINBA peaks. Overlap between called peak sets from ZINBA, MACS, and F-seq for FAIRE were more disparate than those found in high signal-to noise CTCF data (Figure S7b in Additional file 1).

**Figure 6:**
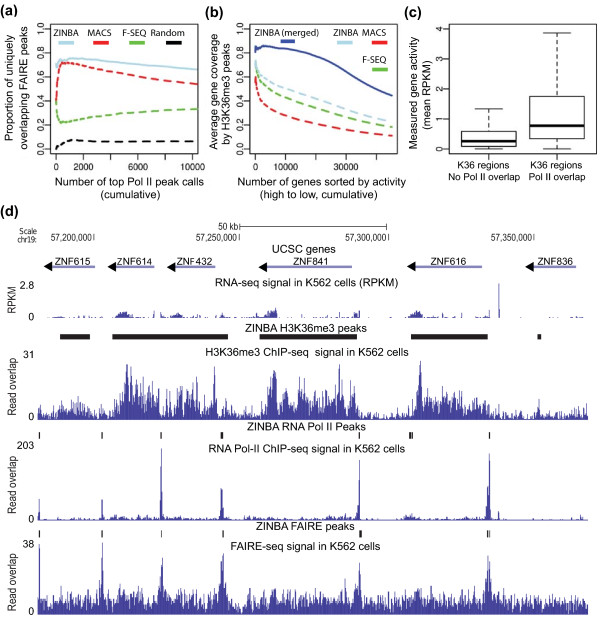
**ZINBA calls broader regions of signal and selects sets of peaks that are coherent across datasets**. **(a) **The proportion of the top cumulative sets of MACS (red dashed line), F-Seq (green dashed line) and ZINBA refined (light blue line) RNA Pol II peaks that uniquely overlap a FAIRE-seq peak called by the respective method. For comparison, overlap was also compared using randomly permuted RNA Pol II and FAIRE-seq ZINBA peak calls (black dashed line). **(b) **The average coverage of the cumulative sets of the top N ranked genes (expression, high to low) by H3K36me3 regions called by MACS (red dashed line), F-Seq (green dashed line) and ZINBA unrefined regions (light blue dashed line). The set of unrefined ZINBA H3K36me3 regions were further clustered throughout the genome to merge nearby peaks (blue solid line) and compared to the ranked list of genes in terms of gene body coverage. **(c) **Comparison of measured gene expression levels for the set of ZINBA H3K36me3 broad regions that either did or did not overlap a ZINBA RNA Pol II broad region. Those overlapping a ZINBA RNA Pol II broad region had three-fold higher median levels of measured gene expression than H3K36me3 regions that did not have any overlap. **(d) **Representative view of the set of H3K36me3 broad, FAIRE-seq refined and RNA Pol II refined ZINBA peak calls displayed in the UCSC Genome Browser along with the respective read overlap data. For reference, the set of genes (top row) and RNA-seq data (second row) are included. RPKM, reads per kilobase per million mapped reads.

Open chromatin regions tend to have strong correspondence to active regulatory elements and promoter regions of expressed genes [40]. Comparison of the set of ZINBA RNA Pol II and FAIRE-seq refined peak calls yielded a significantly higher degree of overlap compared to the other algorithms (Figure 6a), indicating consistency in ZINBA peak calls across data types.

#### ZINBA captures broad patterns of enrichment

The deposition of H3K36me3 is mediated by enzymes that travel along with RNA Pol II during transcriptional elongation, and therefore this histone modification typically occurs in broad segments encompassing a large proportion of gene bodies [41]. Utilizing the 'broad' ZINBA setting (Materials and methods), the H3K36me3-enriched regions identified by ZINBA correspond to the broad patterns of enrichment covering actively transcribed gene bodies, as expected.

On average, 80% of the lengths of the top 'N' most active UCSC gene bodies were covered by the set of H3K36me3 ZINBA peaks (Materials and methods; Figure 6b). A lower level of gene body coverage was found from other methods. Of the 40,180 H3k36me3 merged ZINBA peaks, 71% overlap a gene body, compared with only 59% of F-seq peaks merged in a similar fashion, suggesting higher specificity of these broad ZINBA regions to gene bodies. Of the set of ZINBA merged peak calls that overlapped a gene body, the median and 75th percentile of peak lengths was 5,374 and 18,370 bp respectively, indicative of the broader set of features that are being called (Figure S8 in Additional file 1).

Within the set of H3K36me3 enrichment regions identified by ZINBA, those that overlap ZINBA RNA Pol II broad regions also contain significantly higher levels of RNA expression compared to those that do not overlap broad RNA Pol II regions (Figure 6c). Approximately 85% of ZINBA H3K36me3 broad regions that overlap a ZINBA RNA Pol II broad region contain non-zero RNA-seq signal (7,585 out of 8,873 overlapping regions), compared to only 58% of those that do not (18,134 out of 31,312 non-overlapping regions). Furthermore, of ZINBA H3K36me3 regions with non-zero RNA-seq signal, those that overlapped a ZINBA RNA Pol II broad region had three-fold higher median RNA expression. The relationships we observe among our ZINBA calls recapitulates the biology of H3K36me3, where higher levels RNA Pol II activity correspond to higher levels of RNA transcription and histone modification (Figure 6d).

### ZINBA performs comparably with or without input control data

Comparison of ZINBA peak calls from BIC-selected models considering input as a covariate versus those that do not reveal similar performance in isolating relevant enriched regions. For example, 94% of the CTCF ChIP-seq peaks discovered using a model that included input (Table S1 in Additional file 1) were held in common with a model considering only G/C content, mappability score, and the local background estimate as starting covariates. Recovery of sites overlapping a CTCF motif was also very similar (Figure S9a in Additional file 1). This similarity in performance with and without input extended to the lower signal-to-noise H3K36me3 ChIP-seq data (Figure S9b in Additional file 1). Because of the broad nature of H3K36me3 enrichment, we only considered G/C content and the mappability score as potential covariates in the no-input model. These results demonstrate the ability of ZINBA to distinguish regions of enrichment from background in the absence of input control.

### Modeling enrichment covariates is especially beneficial in low signal-to-noise data

Choosing not to model covariates in the enrichment component (Table S1 in Additional file 1) resulted in almost uniform decreases in model confidence in the classification of 'enriched' windows relative to when enrichment covariates are considered (Figure S10 in Additional file 1). This is especially severe in the low signal-to-noise FAIRE and H3K36me3 dataset (Figure S10a, b in Additional file 1), in contrast to the higher signal-to-noise CTCF data (Figure S10c Additional file 1). In H3K36me3 data, significantly fewer windows in chromosome 22 were classified as enriched over background (posterior probability of enrichment greater than 0.5) when enrichment covariates are ignored. Applying this model genome-wide, we find that 60% fewer windows were called at the default threshold prior to window merging, and post-merging we observe much lower coverage of active gene bodies (Figure S9a in Additional file 1), in contrast to CTCF peaks, which change little as a result of ignoring enrichment covariates (Figure S9b in Additional file 1). These results and the simulated data suggest that utilizing covariates provide an increased discriminatory power for distinguishing background and enriched regions, especially in low signal-to-noise data or when information such as an input control is lacking.

## Discussion

A major challenge in the analysis of genomic experiments that employ NGS technology for detection is the reliable integration of information across a multitude of assays and data types, where such integration would provide a more complete picture of genome-wide cellular regulation. We have developed a statistical framework named ZINBA that addresses these issues by providing a platform that is flexible enough to identify genomic regions of enrichment for a variety of DNA-seq data types and signal patterns. ZINBA can also utilize potentially informative covariates to aid in the classification of genomic regions as likely background, enrichment, or zero-inflated regions.

Application of our approach resulted in the recovery of relevant enriched sites across a wide variety of data types without the need for extensive user input to the analysis procedure. In addition, we show that ZINBA peak calls across different sets of data are highly consistent with known biological processes (Figure 6). Enriched regions can be identified in challenging situations, such as in the absence of input control or within DNA copy number amplifications. In the absence of input control, utilizing other covariates yielded similar results as to when input was used in real data.

Previous studies have commented on the non-random nature of background signal in ChIP-seq data [4,27], and that accounting for this non-randomness can improve modeling of background regions and the ability to detect regions of enrichment. In some peak-finding applications, background models assume signal is completely random, and loci with signal greater than this background are deemed as enriched [2,6]. In datasets where certain covariates have strong effects on background signal, this assumption of randomness is violated. Thus, methods relying on this assumption may result in lower sensitivity and lower specificity to detect enriched sites. Our results suggest that this non-randomness may be due in some part to the effects of certain covariates, and their effects on signal may vary depending on the data being analyzed. We account for this non-randomness by modeling background signal with multiple covariates.

The mixture regression framework used in ZINBA is a natural way to accommodate arbitrary sets of relevant covariates, probe for their relationships with component-specific signal, and account for their effects without the need for user specification of the proportion of background in the sample. One of the inherent advantages of our regression-based approach is that read sample and input read counts do not need to be normalized or scaled, and read coverage is accounted for by each component's regression model. This modeling approach is preferable over normalization procedures that adjust for covariate effects prior to enrichment detection for two reasons. First, these normalization procedures assume the covariates have the same effect on both background and enrichment signals, and thus normalize signal across each region in a similar manner. Second, these procedures cannot naturally account for the effects of multiple covariates simultaneously, which is an inherent feature in a regression framework. It is unknown to the user the impact of such normalization procedures on sensitivity, as the effects of covariates may vary between datasets or between background and enriched regions.

As high-throughput sequencing technology matures, the ZINBA framework can allow for the continued evaluation of existing covariates and the addition of new covariates to model DNA-seq data. Examples of additional potential covariates could be scores for the presence of transcription factor motifs, strand cross-correlation, or local overlap with a particular feature of interest. While not implemented currently, we can easily apply our ZINBA to paired-end reads by assigning a paired-end read to a window if the center of the paired end read belongs to this window.

A major drawback to our approach is the computationally intensive model selection process via BIC. We are currently developing a variable selection procedure based on penalized likelihood that will be able to efficiently select component variables. There are also several general factors that affect all methods regardless of the modeling assumptions used. One such covariate is the sequencing depth of a DNA-seq sample, which is directly related to the sensitivity of the assay to detect enriched sites [14]. Often overlooked, however, is the sequencing depth of the matching input control sample, which typically requires greater sequencing depth to obtain coverage levels similar to the experimental sample.

## Conclusions

Two major challenges in the analysis of DNA-seq data are the diversity in signal patterns that exist across the wide range of possible experiments, and sample-specific issues such as CNV that may further complicate analysis. ZINBA is a flexible statistical framework capable of identifying regions of enrichment across a wide variety of DNA-seq data types, enrichment patterns, and experimental conditions. ZINBA's flexibility in modeling background and enrichment regions with sets of covariates allows for the identification of enriched regions in difficult modeling conditions, such as in datasets with complex local CNVs or lacking a matching input control sample. ZINBA can identify both broad and sharp regions of enrichment, and we demonstrate this capability in differentiating RNA Pol II elongation status. In addition, the statistical framework used is applicable to both high signal-to-noise data such as from CTCF ChIP-seq, as well as to low signal-to-noise data such as from FAIRE-seq. ZINBA produces peak calls that are consistent with known biological patterns, and performs favorably relative to existing specialized methods over a broad range of signal patterns and data types. ZINBA is implemented as a freely available R package.

## Materials and methods

### Datasets and model parameters

All data were produced by members of the ENCODE Consortium [31,32] and downloaded as aligned reads (tagAlign) from the UCSC Genome Browser. The FAIRE-seq, RNA Pol-II ChIP-seq, and H3K36me3 ChIP-seq datasets were derived from K562 cells while the CTCF ChIP-seq dataset was derived from GM12878 cells. For data access and generation methods see Additional file 2. The MCF-7 FAIRE-seq data are not yet available through ENCODE, but access to the relevant portions of the data can be found in Additional file 2. All data were analyzed within ZINBA using 250-bp windows and an additional offsets of 125 bp. The set of covariates and all possible pair-wise and three-way interactions were evaluated using the BIC, with the best scoring model formulation being selected for subsequent downstream analyses. For the RNA Pol II, CTCF, and H3K36me3 ChIP-seq datasets we considered G/C content, mappability score, and input control as starting covariates in our model selection procedure unless stated otherwise. For FAIRE, we only considered G/C content, mappability score, and the local background estimate.

### ZINBA step 1: data preprocessing

#### Calculation of signal values

Raw NGS data are composed of millions of relatively short (25 to 75 bp) reads aligned to a reference genome sequence. A sequence read often does not represent the entire DNA fragment recovered with a given assay, but instead one or both ends of the fragment. Therefore, for single-end reads we attempt to approximate the center of each DNA fragment by extending the coordinates for each aligned read in the 3' direction to the average fragment length. For each ChIP-seq dataset, we used an average fragment length of 200 bp, and 134 bp for our FAIRE-seq dataset. The average fragment can be either specified by the user or estimated from the data directly using the cross-correlation function [14] implemented in ZINBA. All subsequent references to a read refer to the extended coordinates, and raw reads refer to the original coordinates.

The genome-wide set of reads is summarized as the count of reads within a set of contiguous non-overlapping windows. Each read is assigned to a single genomic position based on the position of the central base. To avoid bisecting a potentially significant region, a similar set of contiguous non-overlapping windows can be produced that are offset from the starting position by a user specified distance.

#### Calculation of covariate values

A series of covariates are scored for each window, which include G/C content, mappability score, a local background estimate, and read counts from an input control sample (if available). The G/C content is calculated as the proportion of G and C bases in a given window. The tabulation of window read counts from the input control sample is handled exactly as reads from the experimental sample.

The mappability score is calculated as the proportion of all bases within a window that met the criteria for uniqueness imposed during alignment of the raw reads. Typically, raw reads will only be aligned to positions that are unique throughout the genome. However, in some instances a more relaxed criterion may be used, such as with the FAIRE-seq data where raw reads could be aligned to a position that occurred four or less times throughout the genome. ZINBA implements the mappability software provided by PeakSeq [28] to calculate for each base pair the number of times a given k-mer (36 bp) starting at that base occurs throughout the genome. If a base pair receives a score of 1, then only one occurrence of the given k-mer exists throughout the genome and would be a mappable position under the absolute uniqueness criteria. Whereas if a base pair received a score of 5, then it would not be considered mappable for either the uniqueness or the relaxed criteria described above. Before the mappability scores are summarized into the windows, those bases that meet the specified criteria are assigned a new score of 1, while those that do not are assigned a new score of 0. Finally, since the central position of each extended read is used for window assignment, the mappability data are shifted in the same way, where for each base the score of 0 or 1 is shifted both plus and minus one half the average fragment length. As a result, each base in the genome has a score of 0, 1 or 2 depending on whether neither, one or both of the up- and downstream base pairs were mappable, respectively. The sum of mappability scores is tabulated and divided by two times the window size to derive the proportion of mappable bases in the window.

The local background estimate aims to roughly approximate large-scale fluctuations in background signal resulting from local variations in genomic copy number. It is calculated using a sliding window approach where, by default, 100-kb windows are stepped every 2.5 kb across each chromosome. The size of these large windows was selected to be sufficiently large to prevent sites of enrichment from influencing the estimate, but small enough to preserve enough resolution to capture local fluctuation in background signal. The number of reads per mappable base pair is calculated for each window. Windows that span the boundaries of CNVs are problematic, resulting in artificially inflated and deflated estimates of local background. Therefore, an additional step is employed to identify these change points and any windows straddling these boundaries are removed (Additional file 2). For each ZINBA window, which is considerably smaller than 100 kb, the local background estimate is computed as the average number of reads per mappable base for all overlapping 100-kb windows, multiplied by the length of the ZINBA window.

### ZINBA step 2: data analysis

Because of the discrete nature of window read counts, this summary of the data can be modeled by either the Poisson or the negative binomial distribution. The negative binomial distribution can be considered as an extension of Poisson distribution to handle over-dispersion, that is, the situation where the variance of the counts is larger than expected by a Poisson distribution. Instead of using a single Poisson or negative binomial distribution, we find that our approach of modeling DNA-seq data by a mixture of negative binomial distributions provides an adequate representation of the data (Figure S11 in Additional file 1)

#### Mixture regression to select enriched regions

The mixture regression model is implemented using an EM algorithm [29] that consists of four major steps: initialization, expectation-Step, maximization-Step, and convergence checking. Given a model file from ZINBA step 1, windows that meet a user-defined enrichment posterior probability threshold are selected in the following manner (mathematical details are given in Additional file 2).

##### Model initialization

Initialization of the EM algorithm is the assignment of initial component memberships for each window. Each window has an associated posterior probability vector (*τ*_*i*0_, τ_*i*1_, τ_*i*2_) describing its posterior probability of belonging to each component, where for window *i*, 0 corresponds to the zero-inflated component, 1 corresponds to the background component, 2 corresponds to the enrichment component, and *τ*_*i*0_, *τ*_*i*1_, *τ*_*i*2_= 1. To initialize the model parameters, we create several starting partitions of the data and use these partitions to determine the initial values of model parameters for each component. Under the assumption that enrichment signal is generally larger than background signal, windows with largest window read counts are assigned to the enrichment component such that (*τ*_*i*0_, *τ*_*i*1_, τ_*i*2_) = (0,0,1). Multiple starting partitions are generated such that 0.1%, 1%, 5%, 10%, and 15% of the largest windows in terms of read count are assigned to the enrichment component, and all other windows with non-zero window read counts are assigned to background. All windows with zero read counts are assigned to the zero-inflated component. For each partition we run the EM algorithm multiple times cycling between the E and M steps and choose the partition that provides the best fit.

##### M-step

In the maximization-step (M-step), we apply weighted GLMs to each component, where *τ*_*i*0_, *τ*_*i*1 _and *τ*_*i*2 _are used as the weights of the *i*^th ^window in the corresponding component. These weights represent current knowledge of the probabilistic classification of a window into each component and are updated in the E-step. Window counts in the enriched and background components are modeled using weighted negative binomial regression, allowing for over-dispersion in the distribution of window read counts.

The prior probability that a window is zero-inflated *π*_*i*0_is directly modeled using weighted logistic regression. By using *τ*_*i*0_, *τ*_*i*1_, and *τ*_*i*2_as regression weights for the zero-inflated, background, and enriched regression models, respectively, we are able to partition the same window count across three regression models in a manner proportional to the likelihood that it belongs to each component. For example, windows with a zero-inflation weight of 0, background weight of 1, and enrichment weight of 0 will contribute only to the estimation of the background regression model. Each weighted GLM is maximized using the iteratively reweighted least squares algorithm. At the end of this step, we have estimates of the component-specific covariate effects and the proportion of data belonging to the background and enrichment components (denoted by *π*_1 _and *π*_2_, respectively). These two proportions are derived from *τ*_1 _= *τ*_11_, *τ*_21_,... *τ*_*n*1_and *τ*_2 _= *τ*_12_, *τ*_22_,... *τ*_*n*2_, the vector of window posterior probabilities corresponding to enrichment and background, respectively.

##### E-step

In the Expectation-step (E-step), we update the posterior probability of component membership *τ*_*i*0_, *τ*_*i*1_, and *τ*_*i*2_for each window given the regression estimates from the M-step. Given the read count of a window *y_i _*and an associated covariate vector *X_i_*, we update (*τ*_*i*0_, *τ*_*i*1_, τ_*i*2_) at iteration *k *such that:

where

and

is the indicator function that *y_i _*is equal to zero. Also:

corresponding to the negative binomial distributions for the background component (*j *= 1) and the enrichment component (*j *= 2). The predicted mean for each window  is dependent on the estimate of the component-specific covariate effects , the set of component-specific covariates for window *i X_ij_*, and  is the estimate for the dispersion parameter in component *j*. The value  is the prior probability that a window is zero-inflated given the zero-inflated covariate estimates *γ*^(*k*) ^from the M-step and associated set of covariates *X*_*i*0_(Additional file 2). Mixture proportions  and are defined as before for iteration *k*. Each posterior probability *τ*_*i*0_, *τ*_*i*1_, and *τ*_*i*2_can be thought of as the weighted likelihood that window *i *belongs to component *j *given a windows signal value, set covariates, and the estimated component-specific covariate relationships with average signal in each component. Because enrichment makes up a small proportion of the genome, enrichment covariates generally have a smaller role in influencing classification because of the smaller enrichment proportion *π*_*i*2_. In iteration *k *+ 1,  are again used as regression weights for each component's weighted GLMs.

##### Convergence

The algorithm cycles between the E-step and the M-step until the absolute change in model log likelihood from 10 iterations prior is less than 10^-5^. Windows with posterior probabilities of enrichment greater than a user-specified threshold are considered to be enriched. By default the threshold is 0.95, although this can be lowered to 0.5 if needed. Windows with enrichment posterior probabilities close to 0.5 have ambiguous membership, although the majority of these probabilities for BIC selected models tend to be either close to zero or one (Figure S11b in Additional file 1).

In this modeling framework, there is an inherent assumption of the independence of neighboring windows; however, in real data, correlations between nearby windows are to be expected. This correlated structure is most similar to those found in the analysis of a time series of discrete counts, where neighboring counts are correlated in a serial fashion. More complicated models, such as hidden Markov models and related methods incorporating covariates, would be computationally intensive to implement given the model's complexity [42] and the size of each chromosome. With the default window size, the number of observations for the smallest chromosome is nearly 134,000 windows. One reasonable assumption of the correlation structure is a symmetric autoregressive structure such that the correlation between the *i*^th ^window and the *l*^th ^window is *r*^|*i-l*|^, where *r *is the correlation parameter to be estimated. Under this assumption, previous studies have shown that ignoring local correlation leads to smaller standard errors of covariates but has little effect on the estimated covariate effects themselves in GLMs [43,44]. Therefore, we do not expect correlation to have a significant impact on covariate estimates; however, we still only discuss covariate effects in a qualitative fashion.

##### Selecting relevant covariates using BIC

Selecting the most optimal set of model covariates to achieve the best classification outcome and model fit is not a trivial task. ZINBA uses the BIC [30] to choose the most parsimonious set of covariates that best explains the variation seen within each mixture component. A typical set of covariates includes the mappability score, G/C content, and input control. When sequencing data from an input control sample are not available, the local background estimate can be considered to control for local fluctuations in copy number or other changes in local chromatin structure. Higher order pair-wise and three-way interaction terms are also included between covariates when their lower order effects are in the model. For example, a pair-wise interaction term between G/C content and mappability score will not be considered if any one of these two covariates is not included in the model. Due to the computational cost, the BIC calculations are performed on a restricted set of chromosomes. While the resulting model fit may not be optimal, ignoring interaction terms between starting covariates greatly reduces the number of models to be computed under the BIC procedure. This results in faster processing and does not adversely impact the recovery of relevant peaks (Figure S12 in Additional file 1). We also provide an additional heuristic that further reduces the number of models considered by only considering covariates in the enriched and background components during model selection. With this heuristic and considering three starting covariates with interaction, model selection takes approximately 4 hours for FAIRE data using eight 2.8 GHz Intel Xeon processors (361 models considered).

### Step 3: peak boundary refinement

#### Isolating exact peak boundaries within merged regions

Following analysis using the mixture regression model, overlapping or adjacent windows with an enrichment probability greater than a user-specified threshold (default 0.95) are merged to form significant regions. ZINBA employs a shape detection algorithm to analyze the higher-resolution read overlap data (single base pair count) within each significant region to identify and refine the boundaries of potential punctate enrichment sites. This sequential detection of broader regions and then punctate regions within broader regions allows for more flexibility in detecting various enrichment patterns.

The shape detection algorithm consists of two steps. First, the set of local maxima within the merged significant region is identified. Second, the boundaries of punctate enrichment sites surrounding these local maxima are determined. Specifically, local maximums are determined using a modified matrix-based algorithm from the massspecwavelet package in R. Local maxima greater than a user-defined quantile threshold are retained for boundary refinement. The boundaries are determined using best linear fit for the read overlap data on either side of each maximum. For each side, a simple linear model is fitted originating at the maxima and extending to the base pair position that maximizes the R^2 ^of the linear model. Any local maxima within N bp of each other or whose boundaries overlap are merged into a single peak, where N is 100 bp by default. The set of chromosomal coordinates for each refined region is returned along with the position of the local maxima, the single base pair count score at the maxima, and the maximum posterior probability of the original windows within the merged region.

In general, peak refinement is most useful when one expects a mix of punctate peaks within broader regions, as in RNA Pol II data (Figure S13 in Additional file 1), where ZINBA peak refinement improves the results of other software. In CTCF ChIP-seq, refinement does not make much of a difference, as expected, and in FAIRE, ZINBA still performs favorably to other methods. In addition, ZINBA's peaks have much more favorable correspondence across data types in relation to ZINBA-refined peaks from other software (Figure S13d in Additional file 1).

### Simulation of data

Zero-inflated, background, and enriched window counts are simulated using a two-step procedure. In the first step, background and zero-inflated window counts are randomly simulated given a set of pre-specified covariates and their effects. A random subset of background windows is selected to be enriched and the read counts of these are adjusted according to conditioning on the selected covariates for enriched windows if desired.

Let (*X_z_, X_B_*) be covariate matrices for the zero-inflated component and background component. The number of rows in each matrix is the total number of windows sampled and the number of columns is equal to the number of chosen covariates plus the intercept. The covariates could include, for example, mappability score, G/C content, local background, and read counts from an input control sample in each window. According to the modeling assumptions in Additional file 2, the predicted background mean count of a window is given as  and the probability that a window will be zero-inflated is , where  are pre-specified covariate estimates for the zero-inflated and background components, respectively. These pre-specified parameters are taken from a previous real data analysis in order to simulate realistic background counts.

For the vector of calculated background window means *μ_B _*and dispersion parameter , a set of window counts is simulated using the negative binomial distribution in R. A window is randomly selected to be zero-inflated with probability *π_i, z_*, where windows selected as zero-inflated are set to 0. To simulate enriched windows, the desired proportion of background windows are randomly selected as enriched. To incorporate relationship between the set of enriched windows and covariates, such as with G/C content, the count for enriched window *i *is simulated such that *Y_i, sim _*~ *NB*(*μ *= *b × GC_i _*+ *a, θ_E_*), where *Y_i, sim _*is a random count from the negative binomial distribution with mean value *b × GC_i _*+ *a*, and over-dispersion parameter of enrichment *θ_E_*. The signal-to-noise ratio and the strength of the G/C content covariate effect on enrichment counts can be tuned by altering parameters *a *and *b*. For example, when *b *is 0 there is no relation between window read counts and G/C content; otherwise, the sign of *b *determines whether G/C content is positively or negatively related to window counts.

### Calculation of RNA pol II stalling score

For ZINBA RNA Pol II peaks within 1 kb of genes with non-zero gene expression, we calculate an RNA Pol II 'stalling' index for each peak such that:

where *Length_i, punctate _*is the length of the punctate peak found within a broad ZINBA peak region, *Length_i, broad _*is the length of the broader ZINBA peak, *MAX*(*Height_i, punctate_*) is the maximum read overlap height corresponding to the punctate peak, and *Median *(*Height_i, broad_*) is the median read overlap height of the broader region excluding the punctate peak. A pseudocount of 1 was added to the numerator and denominator of the height ratio to avoid dividing by zero. To determine the relationship between the stalling score and gene expression (in RPKM (reads per kilobase per million mapped reads)), a median regression line (robust to outliers) was fit to the natural log of the gene expression data using the stalling score as a covariate. To better capture broad regions, we merge enriched windows within 5 kb and then applied the ZINBA peak refinement to these broad regions to obtain the punctate sites within.

### Assessing performance across peak calling algorithms and datasets

ZINBA, MACS and F-Seq were run using the default set of parameters with the goal of calling at least 50,000 peaks. Running MACS on FAIRE-seq data without an input control sample required the mfold parameter to be lowered to 10 and the *P*-value threshold increased to 0.001 to generate enough peaks. For the RNA Pol II ChIP-seq data the ZINBA posterior probability threshold had to be lowered to 0.5 to generate enough peaks.

The set of ranked peaks for each algorithm was compared to a set of biologically relevant features. Ranked peaks were used because there was not a straightforward way to impose a single threshold for all algorithms due to differences in the assessment of significance (MACS uses *P*-values, F-Seq uses kernel density estimate, and ZINBA uses posterior probability). Peaks were considered to overlap a biologically relevant feature when the peak center was within 150 bp, which emphasized localization at the set of features. The set of metrics related to overlap, peak length and proximity to features was carried on an increasing cumulative set of the top N ranked peaks. It is common for ZINBA regions called as enriched to have a posterior enrichment probability of 1; therefore, these windows were further differentiated based on the maximum read overlap score.

Overlap between the FAIRE-seq, RNA Pol II and H3K36me3 peaks with the set of active genes (UCSC knownGenes, hg18) was carried out using the intersectBed function in BEDTools [45] with default parameters. The broader set of enriched regions called by ZINBA for the H3K36me3 and RNA Pol II datasets were collapsed by clustering regions within 5 kb (optional) of each other into a single region. To generate a set of random peaks, the shuffleBed function in BEDTools was used to randomize the locations of ZINBA enriched regions, while maintaining localization on the same chromosome and ignoring centrometric regions using the -chrom and -excl options.

Gene activity was measured as the isoform with the maximal value contained within UCSC gene bodies using RNA-seq data (RPKM), where a gene body was defined as from transcription start to stop. Therefore, each gene was assigned a signal score based on this measured value. Then, the average coverage by H3K36me3 peak regions of the set of N most active gene bodies by this measure was calculated using the coverageBed function in BEDTools for each method.

### Software implementation

ZINBA (version 2.0) is implemented as an R package (Additional file 3), and updated versions can be accessed at the software website [46]. The core of the mixture regression framework is implemented in C to improve computational efficiency. ZINBA can be run on a desktop computer with at least 3 GB of RAM and a 2 GHz processor. The software is applicable to any species with a sequenced genome. ZINBA can be run using a GUI (graphical user interface) or from the command line, and is capable of being installed on multi-core computing clusters, allowing for extremely high-throughput capacity. Currently, ZINBA is available for Linux, UNIX, and Mac OSX.

## Abbreviations

BIC: Bayesian information criterion; bp: base pair; ChIP: chromatin immunoprecipitation; CNV: copy number variation; CTCF: CCCTC-binding factor; DHS: DNase hypersensitivity site; FAIRE: formaldehyde-assisted isolation of regulatory elements; GLM: generalized linear model; H3K36me3: histone H3 lysine 36 tri-methylation; NGS: next generation sequencing; RNA Pol II: RNA polymerase II; RPKM: reads per kilobase per million mapped reads; TFBS: transcription factor binding site; TSS: transcription start site; ZINBA: Zero-Inflated Negative Binomial Algorithm.

## Competing interests

The authors declare that they have no competing interests.

## Authors' contributions

NR and PG conceived of the project under the direction of WS, JI, and JDL. NR, PG, WS, JI, and JDL wrote the manuscript. NR, PG, and WS coded the software. All authors read and approved the final manuscript.

## Supplementary Material

Additional file 1 **Figures S1 to S13 and Table S1**. Supplementary Figures S1 to S13, and Table S1, corresponding to parameter estimates from the ZINBA BIC-selected models. Click here for file

Additional file 2 **Supplementary methods**. Mathematical details of the ZINBA mixture regression algorithm, in addition to data access details. Click here for file

Additional file 3 **ZINBA version 2.0**. A freeze of the ZINBA software as of 9 July 2011, which is included in the manuscript for archival purposes only. We recommend that users download ZINBA from our website [46] to ensure that the most up-to-date version is installed. Click here for file
