# Supplementary material for: ZINBA integrates local covariates with DNA-seq data to identify broad and narrow regions of enrichment, even within amplified genomic regions
Source: Genome Biol. 2011 Jul 25;12(7):R67. doi: 10.1186/gb-2011-12-7-r67 (PMC3218829; doi:10.1186/gb-2011-12-7-r67)
Supplement: Additional file 1 — Figures S1 to S13 and Table S1. Supplementary Figures S1 to S13, and Table S1, corresponding to parameter estimates from the ZINBA BIC-selected models. [file gb-2011-12-7-r67-S1.PDF]

Figure S1. Application of Model 1 to simulation results where there is a negative association of G/C-content with simulated enrichment corresponding to Figure 2.

Heat maps of ZINBA-assigned enrichment posterior probabilities from Model 1 are laid over the simulated data to illustrate the pattern in enrichment classification when covariates are not utilized to model each component of the data. Pink colors pertain to highly significant windows called by the model and dark blue pertains to the least significant windows. Enrichment classification in this model ignores informative trends in background and enrichment with G/C-content, and classifies windows simply by signal. As a result, **Figure 2C,D** shows the decreased performance of this model relative to Model 3, which correctly incorporates the simulated relationships of G/C-content in each component. Results are shown for **(A)** High signal-to-noise simulated data and **(B)** low signal-to-noise simulated data.

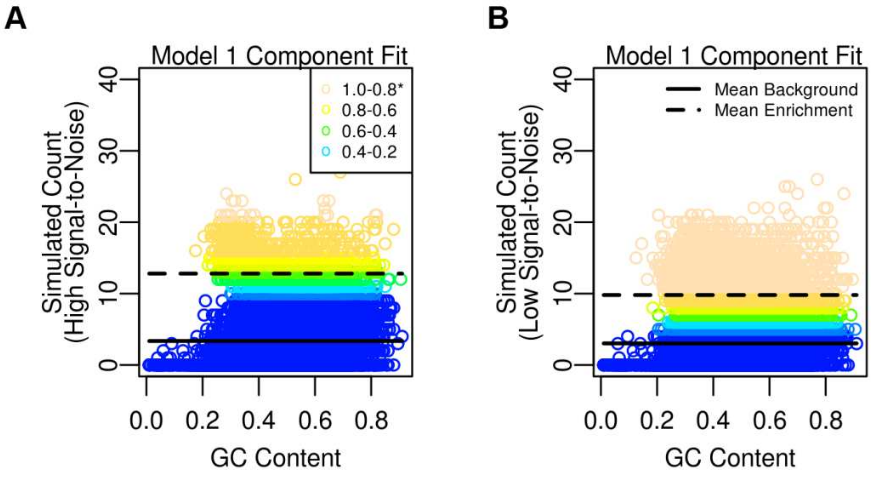

Figure S2. Simulation results where there is no association between signal in the enrichment component and G/C-content over a variety of conditions.

Simulated data corresponding to **(A-B)** high signal-to-noise ratio and small proportion of enriched windows, **(C-D)** moderate signal-to-noise ratio and moderate proportion of enriched windows, and **(E-F)** low signal-to-noise ratio and high proportion of enriched windows. The raw data for each simulation is shown in the left column, where the y-axis is the simulated count in a window and the x-axis is the corresponding G/C-content in the window. The set of enriched sites are represented as black circles while the density of background windows are shown in blue. The right column shows ROC curves of different model formulations, including no covariates used for the components (Model 1), G/C-content only used as a covariate with the background and zero-inflated component (Model 2) and G/C-content used for the background, zero-inflated and enrichment components (Model 3). All models perform similarly, although we see a slight advantage of Model 3 and Model 2 because it still models G/C-content's simulated associations in background and zero-inflated regions.

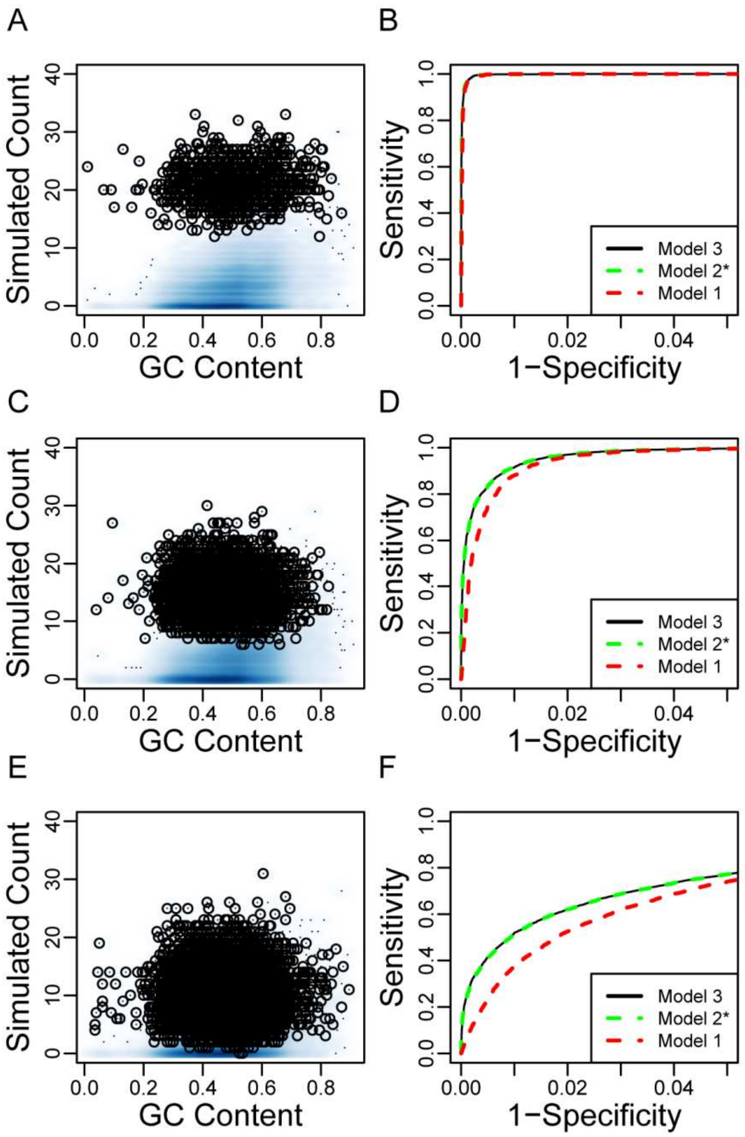

A
B
C
D
E
F
Simulated Count
GC Content
Sensitivity
1−Specificity
Model 3
Model 2*
Model 1

Figure S3. Simulation results where there is a positive association between signal in the enrichment component and G/C-content over a variety of signal conditions.

Simulated data corresponding to **(A-B)** high signal-to-noise ratio and small proportion of enriched windows, **(C-D)** moderate signal-to-noise ratio and moderate proportion of enriched windows, and **(E-F)** low signal-to-noise ratio and high proportion of enriched windows. The raw data for each simulation is shown in the left column, where the y-axis is the simulated count in a window and the x-axis is the corresponding G/C-content in the window. The set of enriched sites are represented as black circles while the density of background windows are shown in blue. The right column shows ROC curves of different model formulations, including no covariates used for the components (Model 1), G/C-content only used as a covariate with the background and zero-inflated component (Model 2) and G/C-content used for the background, zero-inflated and enrichment components (Model 3). As the signal-to-noise ratio decreases, Model 3 has an increasing advantage over Model 2 and Model 1. In the lower signal-to-noise ratio condition, not modeling the relation between G/C-content and enriched windows causes Model 2 to perform worse than Model 1. This is because differences in the effect of G/C-content between the enriched and background components interferes with Model 2's ability to accurately model background and results in poorer classification.

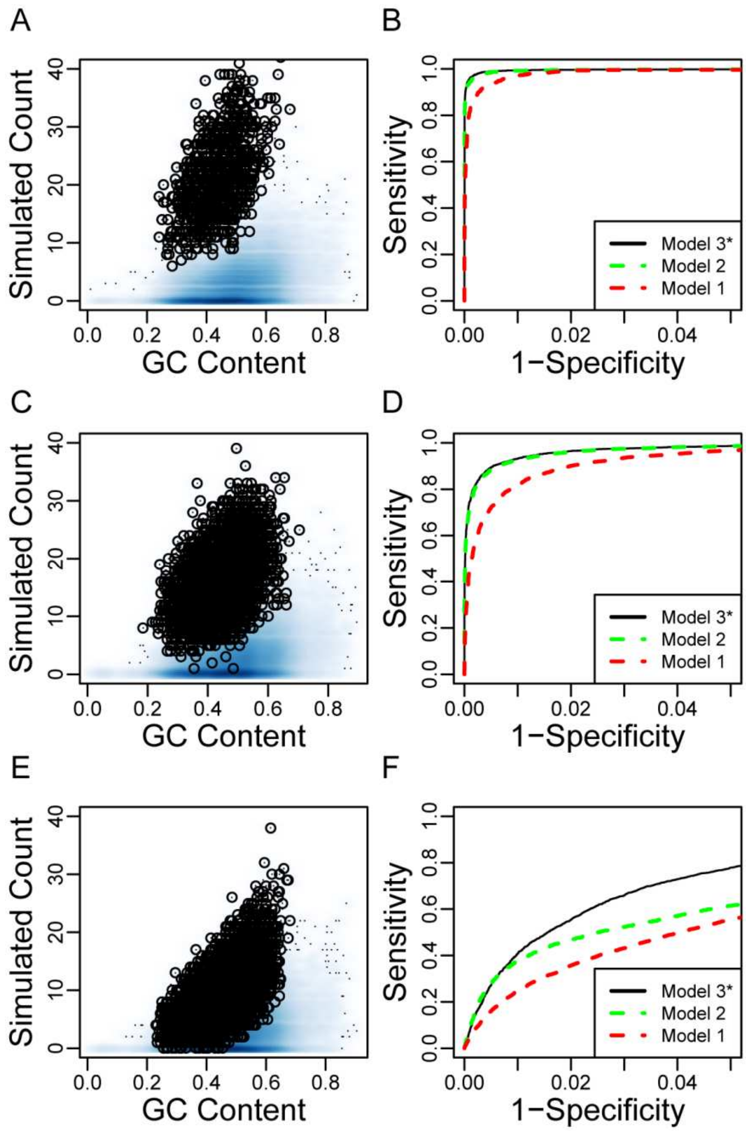
A
B
C
D
E
F
Simulated Count
GC Content
Sensitivity
1−Specificity
Model 3*
Model 2
Model 1

Figure S4. Scatter plots of window read counts versus G/C-content in 250 bp windows (chromosome 22)

The natural log of window read counts from **(A)** K562 RNA Pol II input control sample, **(B)** K562 RNA Pol II ChIP-seq, **(C)** GM12878 CTCF input control sample, **(D)** GM12878 CTCF ChIP-seq, **(E)** K562 H3k36me3 input control sample, and **(F)** K562 H3K36me3 ChIP-seq are plotted against their window G/C-content. The model-estimated background effect of G/C content is plotted for each (with all other covariates fixed at their median values). The relationship of G/C is generally inconsistent between input control and ChIP samples except in the case of H3K36me3, where the effect of G/C content is relatively small in H3K36me3 ChIP-seq background signal.

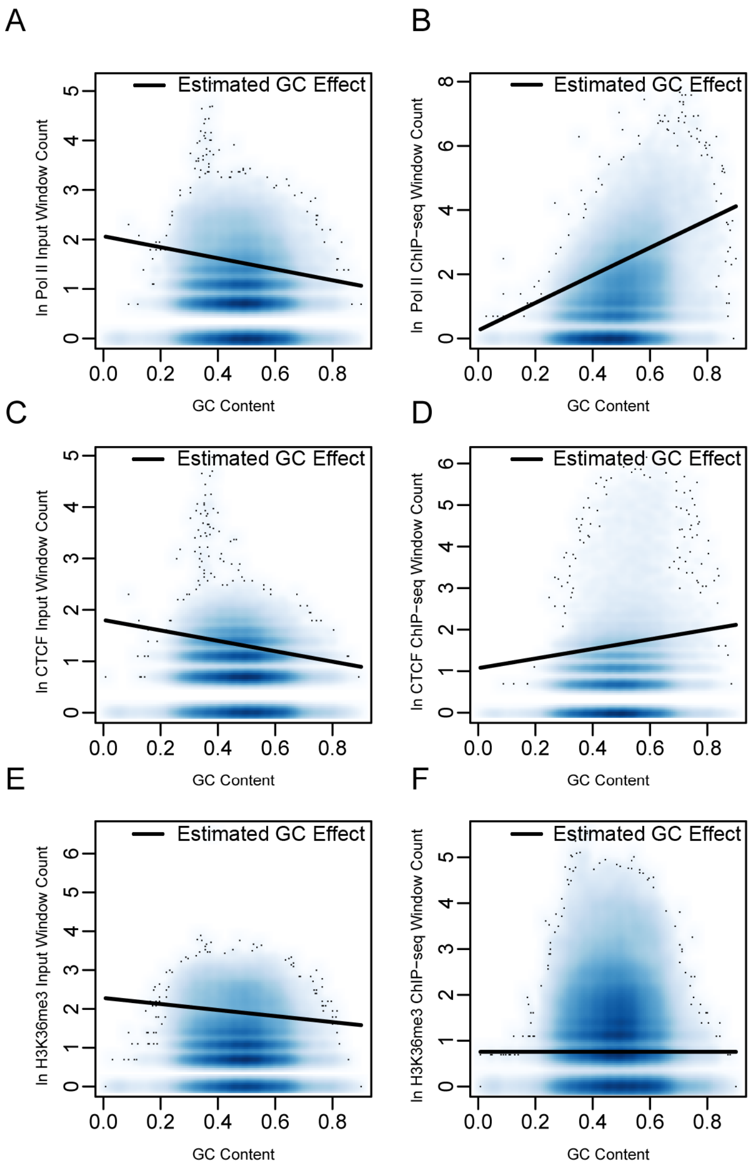

Figure S5. Example of RNA Pol II ChIP-seq region comparing peak calls from F-seq, MACS, and ZINBA. (A) ZINBA's unrefined region is able to capture the broader region of signal, and the refined estimate is able to capture signal specifically localized around the punctate peak. **(B)** Venn diagram showing mutual overlap between RNA Pol II peak calls from ZINBA, MACS, and F-seq. A much lower degree of overlap is observed for peak calls from each method compared to the CTCF dataset.

A

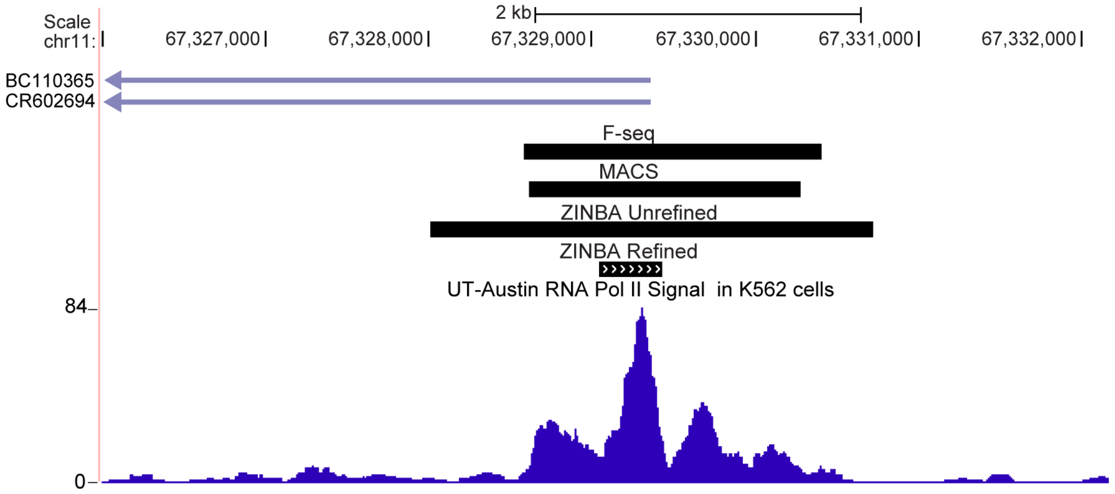

B

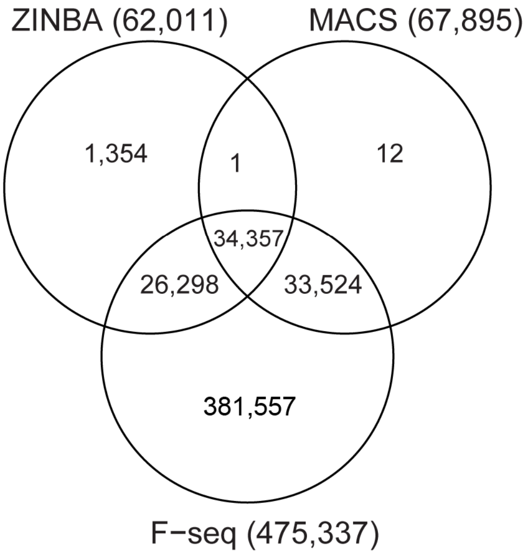

Figure S6. Plot of calculated RNA Pol II "stalling" scores for ZINBA RNA Pol II ChIP-seq peak regions within 1 kb of active genes, versus measured gene expression within the nearby gene body.

(A) Scatter plot of gene expression versus stalling score, considering a stalling metric based only on the height ratio between the punctate peak and the broader region. A median regression line modeling the natural log of nearby gene expression as a function of this stalling score is overlain. **(B)** Scatter plot of gene expression versus the ZINBA stalling score additionally accounting for the ratio of RNA Pol II punctate peak length to broad peak length (**Methods**). A strong negative association can be seen between our stalling score and corresponding expression (p-value < 10^{-10}), where genes having likely stalled polymerase (higher scores) have much lower levels of gene expression. Higher scores are indicative of regions with less elongation but contain a punctate peak near the transcription start site. The score considering only the height ratios of punctate to broad regions explained much less of the variance in measured gene expression (R^2 = 0.04%) versus the ZINBA stalling score (R^2 = 3.5%), suggesting that the incorporation of punctate to broad peak lengths ratios into the ZINBA score represents a marked improvement.

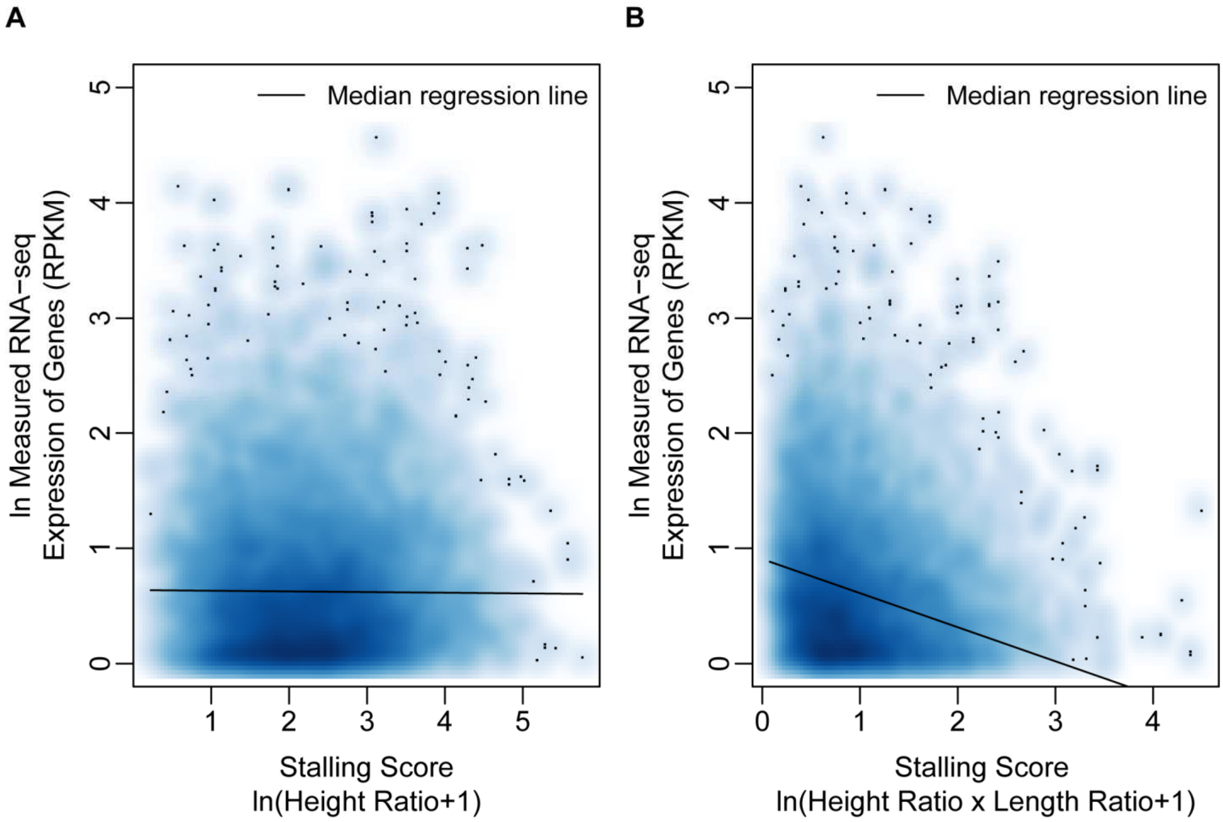

Figure S7. Example of K562 FAIRE-seq signal in an amplified CNV region.
(A) Because input control is not available, the ZINBA BIC-selected model includes our estimate for local background as a starting covariate. ZINBA is able to call regions that are specific to punctate peaks within broader regions of FAIRE signal, while other methods call broader regions in the surrounding regions. FAIRE signal characteristically has greater levels of background that tend to be more pronounced in CNV regions. **(B)** Overlap between peak calls from each method are more disparate compared to results from the CTCF ChIP-seq dataset, indicative of the challenging conditions for peak calling in FAIRE-seq data.

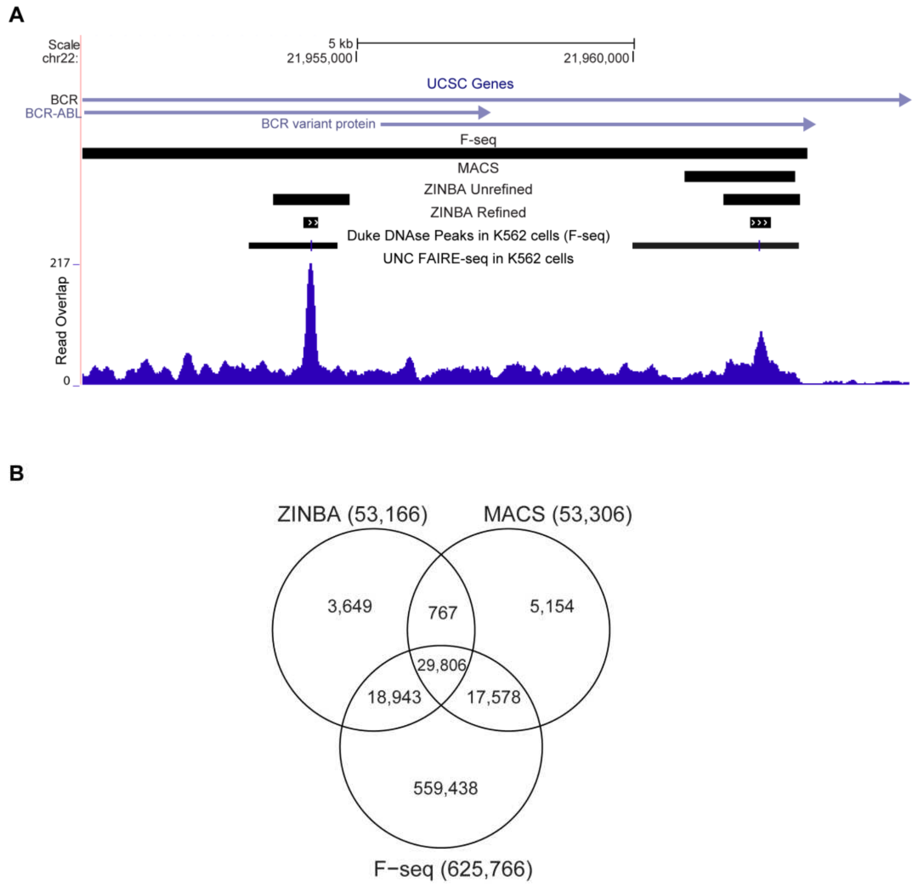

Figure S8. Comparing the distribution of peak lengths corresponding to ZINBA H3k36me3 called regions. Box plot of the distribution of peak lengths from ZINBA H3K36me3 regions reveal broader regions of signal being recovered. In addition to specificity to gene bodies, these regions have a high degree of coverage with active gene bodies (**Figure 6B**) and contain higher levels of gene expression when overlapping a RNA Pol II broad peak (**Figure 6C**)

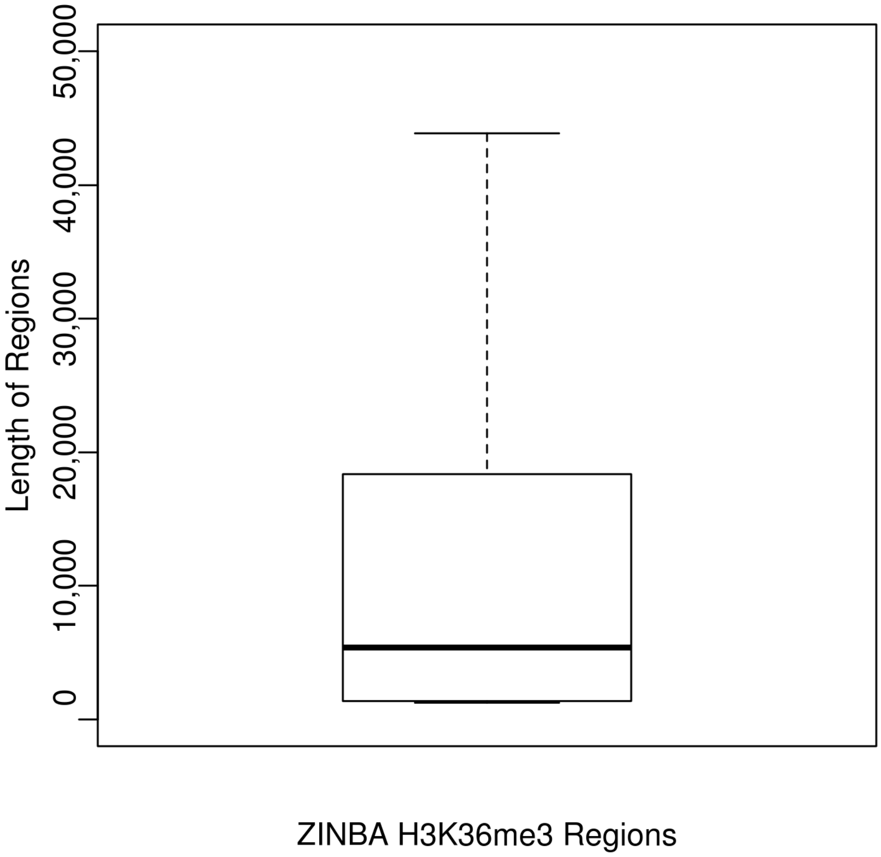

Figure S9. Performance comparison of ZINBA models under different model formulations **(A)** In CTCF ChIP-seq data, BIC selected models not considering input control as a starting covariate (using G/C-content, mappability score, local background estimate) perform similarly to BIC selected models considering input control (using input control, G/C-content, mappability score). In addition, we find that not modeling enrichment covariates has little impact on eventual classification performance (light blue). **(B)** In contrast, not modeling enrichment in low signal-to-noise H3K36me3 ChIP-seq data has a large impact on ZINBA's ability to recover enriched regions spanning gene bodies (light blue). Similar to CTCF, not considering input control (G/C-content, mappability score) results in similar performance as when input control is considered (yellow).

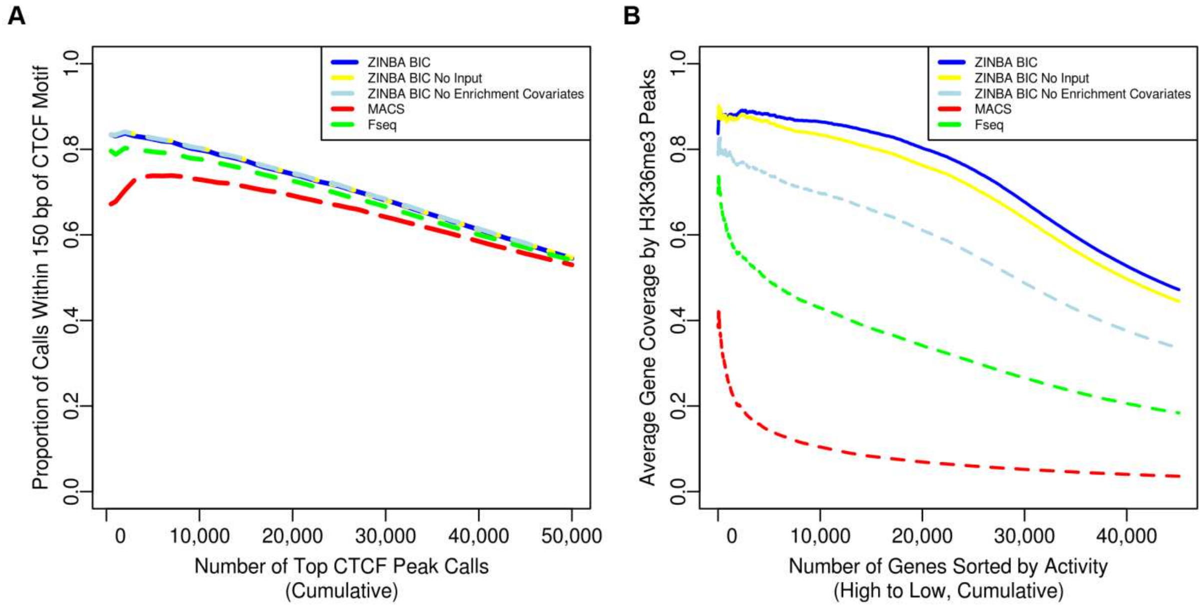

Figure S10. Scatter plots of enrichment classification of BIC selected models vs. BIC selected models lacking enrichment covariates in human chromosome 22.

(A) FAIRE-seq and **(B)** H3K36me3 ChIP-seq represent low signal-to-noise datasets where enrichment is often difficult to distinguish from background. In these datasets a strong decrease in the number of windows classified as enriched is observed when enrichment covariates are ignored in the BIC models from **Table S1 in Additional File 1**. This is in contrast to high signal-to-noise **(C)** GM12878 ChIP-seq, where a much smaller decrease in enrichment classification is observed when enrichment covariates are ignored, as signal alone is sufficient in classification. The red line indicates absolute agreement in posterior probabilities of enrichment from BIC selected models and a corresponding model lacking enrichment covariates.

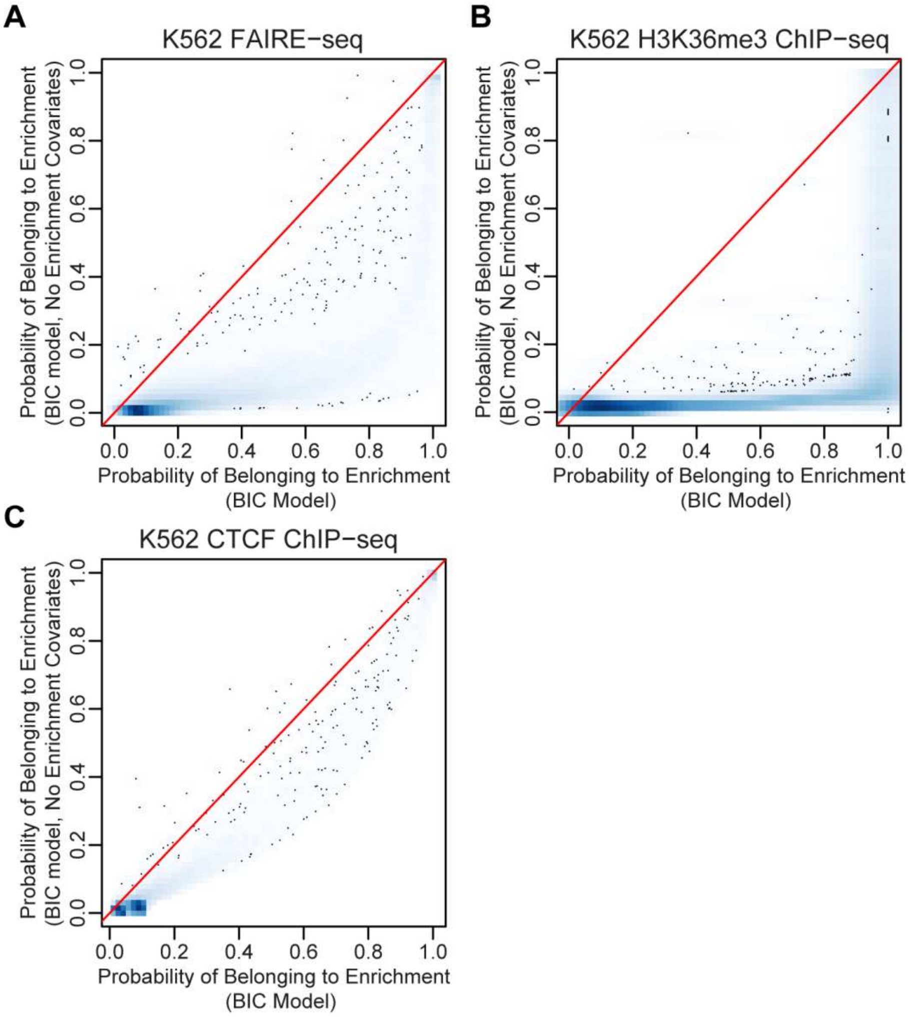

Figure S11. ZINBA mixture regression model fit and posterior probability distribution
(A) Comparison of model fit between ZINBA's mixture regression framework versus traditional Poisson and Negative Binomial regression models on K562 chromosome 22 FAIRE-seq data. To avoid bias in the fitting of background regions, some methods remove likely enriched regions and fit a model on the remaining counts. The drawback of such an approach is that the proportion of enrichment is unknown *a priori*, and thus it is not known how much data to remove or what windows are enriched to begin with. **(B)** Histogram of enrichment posterior probabilities applied to K562 FAIRE-seq chromosome 22 data. Distribution of these probabilities show that ambiguous assignment is rare (probabilities near 0.5)

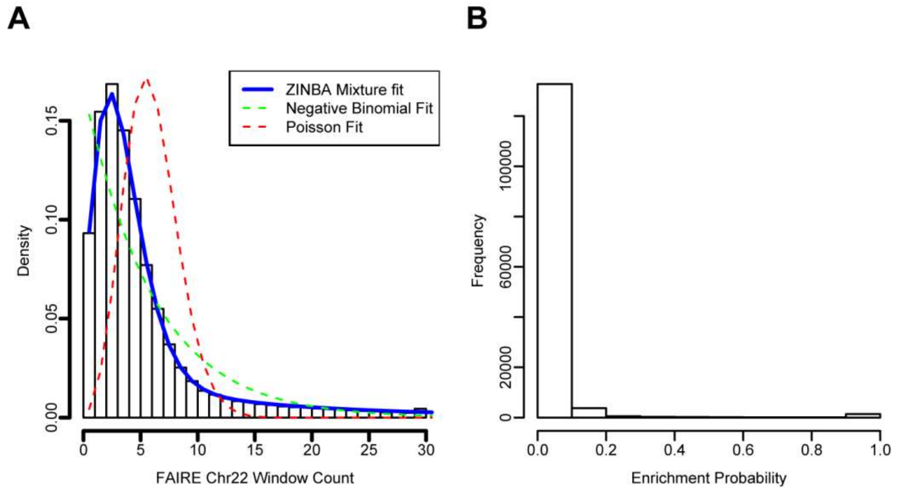

Figure S12. Comparison of peak calling performance in selected ZINBA models when interaction is considered versus when it is not

Recovery of relevant FAIRE peak regions from the ZINBA model using in **Figure 5 D-F** was compared to peaks selected by the ZINBA model selection procedure not considering two and three-way interactions. The resulting peak calls are very similar, suggesting that ignoring interaction does not adversely impact peak calling performance. Considering higher order interactions during the ZINBA automated model selection procedure greatly increases computational time, so considering main effects of three starting covariates reduces the number of models to consider for each component from 19 to 8.

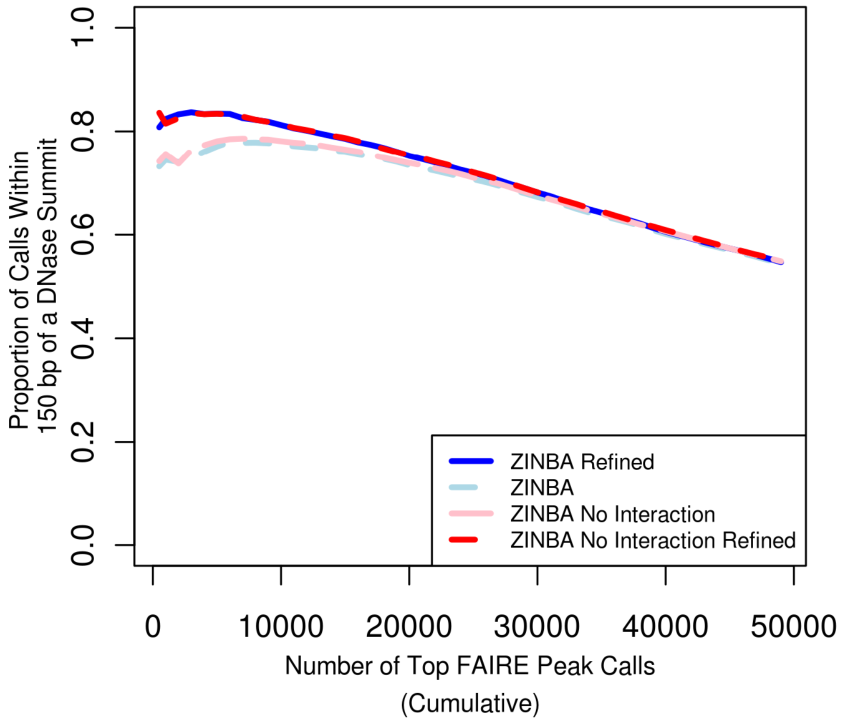

Figure S13. Application of ZINBA peak refinement to MACS and F-Seq regions
Application of peak refinements to regions called in **(A)** CTCF ChIP-seq (corresponding to Figure 5A), **(B)** RNA Pol II ChIP-seq (corresponding to Figure 5D) **(C)** and FAIRE by each method (corresponding to Figure 5F). Peak refinement is most useful in situations where on expects a mixture of punctate and broad regions. As expected, peak refinement as little effect in punctate CTCF ChIP-seq data, and has significant impact in RNA Pol II data. In FAIRE-seq data, we find that ZINBA performs favorably to other methods. **(D)** The overlap between refined FAIRE and RNA Pol II peaks from each method; compare to Figure 6A. Without peak refinement, ZINBA performs well relative to other methods (Figure 5).

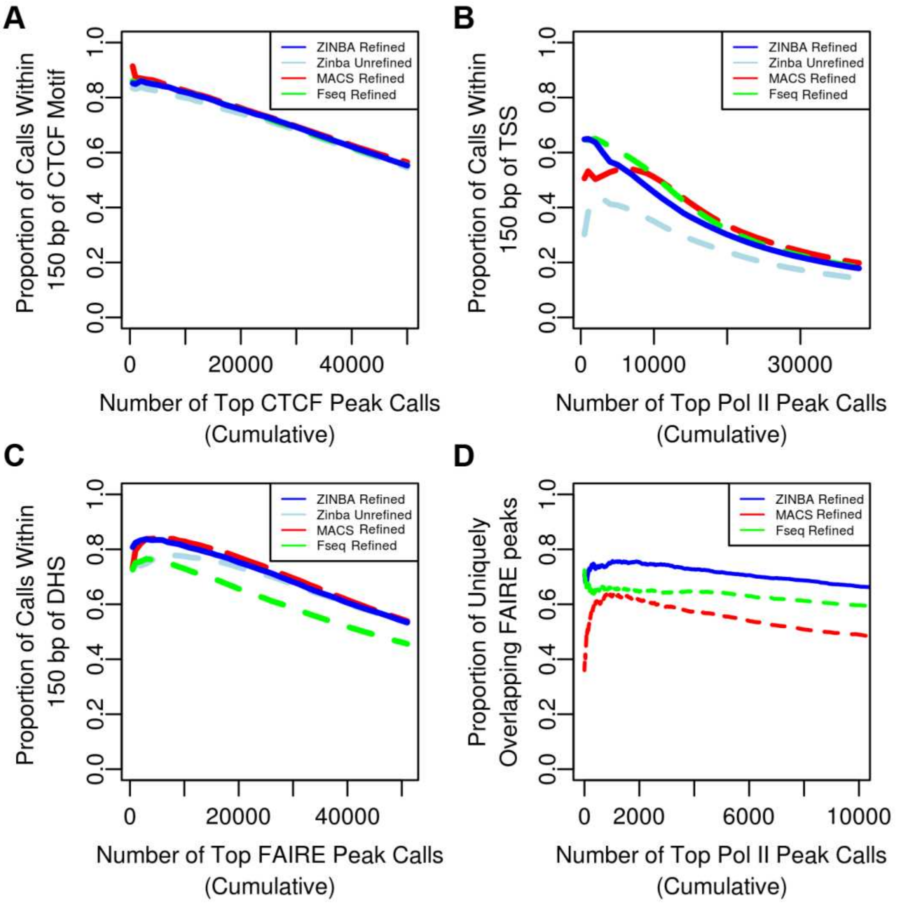

Table S1: Parameter Estimates for BIC selected models for ENCODE DNA-seq datasets (Human Chr 22).

Parameter estimates are given below for BIC-selected covariates. Interaction terms are denoted by ":" in between the interacting covariates. For the background and enrichment components, the estimates are given in terms of the change in log mean count for a one unit increase in that standardized covariate. For the zero-inflated component, the estimate is given in terms of the change in log odds of being zero-inflated for each unit increase in that standardized covariate.

Dataset	Component	Covariate	Estimate
**GM12878 CTCF**			
	**Background**	(Intercept)	-0.0897
		G/C-content	0.2601
		Mappability Score	0.3286
		Input	0.1632
		G/C-content:Input	0.0373
	**Enrichment**	(Intercept)	1.6229
		G/C-content	0.8558
		Mappability Score	0.6737
		Input	0.5003
	**Zero-inflated**	(Intercept)	-3.2968
		G/C-content	0.7713
		Mappability Score	-0.6075
		Input	-0.0791
		G/C-content:Mappability Score	0.1233
**K562 RNA Pol II**			
	**Background**	(Intercept)	0.8890
		G/C-content	0.5758
		Mappability Score	0.3005
		Input	0.5048
		G/C-content:Input	0.1071
	**Enrichment**	(Intercept)	2.0064
		Mappability Score	0.3397
		G/C-content	0.9187
		Input	0.5347
		Mappability Score:Input	0.0962
		G/C-content:Input	0.0867
	**Zero-inflated**	(Intercept)	-1.7309
		Mappability Score	-0.1419
		G/C-content	-0.7708
		Input	-0.3327
		Mappability Score:G/C-content	-0.2179
		Mappability Score:Input	-0.1686
**K562 FAIRE**			
	**Background**	(Intercept)	-1.5322

		G/C-content	-1.4908
		Mappability Score	1.7753
		Local Background	0.9706
		Mappability Score:Local Background	0.1552
	**Enrichment**	(Intercept)	-2.7483
		G/C-content	2.0861
		Mappability Score	1.4268
		Local Background	0.5070
		Mappability Score:Local Background	0.8581
	**Zero-inflated**	(Intercept)	-13.6561
		G/C-content	15.6943
**K562 H3K36me3**		(Intercept)	0.6641
	**Background**	Mappability Score	-0.1383
		G/C-content	0.0547
		Input	0.4589
		Mappability Score:G/C-content	-0.0278
		Mappability Score:Input	0.1173
	**Enrichment**	(Intercept)	1.9265
		Mappability Score	0.2342
		G/C-content	0.0223
		Input	0.5233
		Mappability Score:G/C-content	0.0578
		Mappability Score:Input	0.0540
		G/C-content:Input	0.0137
	**Zero-inflated**	(Intercept)	-37.1736
		Mappability Score	67.0922
		G/C-content	0.8063
		Input	-0.4056
