# Supplementary material for: ZINBA integrates local covariates with DNA-seq data to identify broad and narrow regions of enrichment, even within amplified genomic regions
Source: Genome Biol. 2011 Jul 25;12(7):R67. doi: 10.1186/gb-2011-12-7-r67 (PMC3218829; doi:10.1186/gb-2011-12-7-r67)
Supplement: Additional file 2 — Supplementary methods. Mathematical details of the ZINBA mixture regression algorithm, in addition to data access details. [file gb-2011-12-7-r67-S2.PDF]

Supplementary Methods

Contents

1 Three Component Mixture Regression Framework **1**

1.1 Model Introduction 1

1.2 Derivation of the Complete Likelihood 2

1.3 E-Step 4

1.4 M-Step 5

1.5 Convergence 6

1.6 Robustness of Model Initialization 6

2 Calculation of Local Background Estimates in ZINBA **7**

3 Data Access and Details **8**

4 References **10**

1 Three Component Mixture Regression Framework

1.1 Model Introduction

Let us assume that $Y = (Y_1, \dots, Y_i, \dots Y_n)$ is a vector of n consecutive window read counts from a particular chromosome. We assume Y_i follows a three component mixture distribution consisting of a point mass at zero (corresponding to zero-inflated regions of signal), a negative binomially distributed component (corresponding to background windows), and another negative binomially distributed component (corresponding to enrichment windows). This is an extension of the zero-inflated negative binomial distribution, where we add an additional component to account for stronger signal in enriched windows relative to background windows. Specifically,

$$p(Y_i = y_i \mid \mu_i, \theta, \pi_i) = \begin{cases} \pi_{i0} + (1-\pi_{i0})\pi_1 \left(\frac{\theta_1}{\mu_{i1}+\theta_1}\right)^{\theta_1} + (1-\pi_{i0})\pi_2 \left(\frac{\theta_2}{\mu_{i2}+\theta_2}\right)^{\theta_2} & y_i = 0 \\ (1-\pi_{i0})\pi_1 \frac{\Gamma(y_i+\theta_1)}{y_i!\Gamma(\theta_1)} \left(\frac{\theta_1}{\mu_{i1}+\theta_1}\right)^{\theta_1} \left(\frac{\mu_{i1}}{\mu_{i1}+\theta_1}\right)^{y_i} & \\ +(1-\pi_{i0})\pi_2 \frac{\Gamma(y_i+\theta_2)}{y_i!\Gamma(\theta_2)} \left(\frac{\theta_2}{\mu_{i2}+\theta_2}\right)^{\theta_2} \left(\frac{\mu_{i2}}{\mu_2+\theta_2}\right)^{y_i} & y_i > 0 \end{cases}$$

where $\mu_i = (\mu_{i1}, \mu_{i2})$ correspond to the means of the negative binomially distributed background and enrichment components respectively for window i, and $\theta = (\theta_1, \theta_2)$ are the corresponding dispersion parameters for each

component. Also, $\pi_i = (\pi_{i0}, \pi_1, \pi_2)$ are the corresponding mixture proportions for the zero-inflated, background and enrichment components, respectively. π_{i0} corresponds to the prior probability that window i is zero-inflated, where $\pi_0 = (\pi_{10}, \ldots, \pi_{i0}, \ldots, \pi_{n0})$ is the $n \times 1$ vector of zero inflated prior probabilities for the set of n windows. We set π_1 and π_2 as scalars where $\pi_1 + \pi_2 = 1$. In the next section, we set up an EM algorithm to estimate the maximum likelihood estimates of the model parameters and obtain posterior probabilities of component membership for each window given these model parameter estimates.

1.2 Derivation of the Complete Likelihood

Given this setup, let us write out the complete log-likelihood for the mixture model. The observed data for a chromosome is given as (Y, X_0, X_1, X_2) where

- $Y = n \times 1$ vector of observed window read counts counts
- $X_1 = n \times (p + 1)$ covariate matrix pertaining to the background component
- $X_2 = n \times (q + 1)$ covariate matrix pertaining to the enrichment component
- $X_0 = n \times (r + 1)$ covariate matrix pertaining to the zero-inflation component

where p, q, and r are the number of covariates for the background, enrichment, and zero-inflation components, respectively, and n is the number of windows in that chromosome. For each component we assume an intercept, represented by a column of ones in the first column of each covariate matrix. In the ZINBA data preprocessing step we obtain Y_i and corresponding values of several factors, including window GC content, proportion of mappable bases, read counts from a matching input control (if included) and a local background estimate. We use these factors to construct each of the covariate matrices above, including main effects of each factor and interaction terms between them if desired (pair-wise and three-way).

The missing data in this framework is the true component membership of each window. Let z_{i1} be the indicator function for when window i truly belongs to background, z_{i2} the indicator function for when window i truly belongs to enrichment, and $z_{i0} = 1 - z_{i1} - z_{i2}$ be the indicator function for when window i truly belongs to the zero-inflated component. We consider $z_i = (z_{i0}, z_{i1}, z_{i2})$ to be a draw from the Multinomial distribution such that $z_i \sim \text{Multinomial}(1, (\pi_{i0}, (1 - \pi_{i0})\pi_1, (1 - \pi_{i0})\pi_2))$.

The mean values of the negative binomially distributed background and enrichment components are modelled as a function of a set of covariates through the log link, such that $\log(\mu_1) = X_1\beta_1$ and $\log(\mu_2) = X_2\beta_2$, where μ_1 and μ_2 are $n\times 1$ vectors and X_0, X_1, and X_2 are the covariate matrices pertaining to each parameter. $\beta_1 = (\beta_{01}, \beta_{11}, \ldots, \beta_{p1})$ and $\beta_2 = (\beta_{02}, \beta_{12}, \ldots, \beta_{q2})$ are vector of regression parameters corresponding to the background and enrichment components, respectively. The parameter β_{01} and β_{02} represent the intercept parameter for each component, interpreted as the average level of signal in each component when all component-specific covariates are equal to zero. We also model the vector of prior probabilities for zero-inflation π_0 as a function of a set of covariates through the logit link $\pi_0 = \frac{e^{X_0\gamma}}{1+e^{X_0\gamma}}$, where $\gamma = (\gamma_0, \gamma_1, \ldots, \gamma_r)$ is the vector of regression parameters corresponding to the zero-inflated component. Note that we do not directly model the probabilities of enrichment and background for the sake of robustness of the algorithm, although technically it is straight forward to do so.

Let us derive the log of the complete likelihood for the model given the observed window read counts vector Y and selected covariates. This is derived from the the mixture regression setup in Section 1.2:

$$
\begin{aligned}
L_c(\gamma, \beta_1, \beta_2, z) &= \log\left(\prod_{i=1}^{n} p(Y_i = y_i \mid \mu_i, \theta, \pi_i, z_i)\right) \\
&= \sum_{i=1}^{n} \log\left(p(Y_i = y_i \mid \mu_i, \theta, \pi_i, z_i)\right) \\
&= \sum_{i=1}^{n} z_{i0}\log(\pi_{i0}) + z_{i1}\log(1-\pi_{i0}) + z_{i2}\log(1-\pi_{i0}) \\
&+ z_{i1}\left[\log(\pi_1) + \log\left(\frac{\Gamma(y+\theta_1)}{y_i!\Gamma(\theta_1)}\left(\frac{\theta_1}{\mu_{i1}+\theta_1}\right)^{\theta_1}\left(\frac{\mu_{i1}}{\mu_{i1}+\theta_1}\right)^{y_i}\right)\right] \\
&+ z_{i2}\left[\log(\pi_2) + \log\left(\frac{\Gamma(y+\theta_2)}{y_i!\Gamma(\theta_2)}\left(\frac{\theta_2}{\mu_{i2}+\theta_2}\right)^{\theta_2}\left(\frac{\mu_{i2}}{\mu_{i2}+\theta_2}\right)^{y_i}\right)\right] \\
&= \sum_{i=1}^{n} z_{i0}\log(\pi_{i0}) + (1-z_{i0})\log(1-\pi_{i0}) \\
&+ z_{i1}\left[\log(\pi_1) + \log\left(\frac{\Gamma(y+\theta_1)}{y_i!\Gamma(\theta_1)}\left(\frac{\theta_1}{\mu_{i1}+\theta_1}\right)^{\theta_1}\left(\frac{\mu_{i1}}{\mu_{i1}+\theta_1}\right)^{y_i}\right)\right] \\
&+ z_{i2}\left[\log(\pi_2) + \log\left(\frac{\Gamma(y+\theta_2)}{y_i!\Gamma(\theta_2)}\left(\frac{\theta_2}{\mu_{i2}+\theta_2}\right)^{\theta_2}\left(\frac{\mu_{i2}}{\mu_{i2}+\theta_2}\right)^{y_i}\right)\right] \\
&= L_c(\gamma; y, z) + L_c(\beta_1, \theta_1; y, z) + L_c(\beta_2, \theta_2; y, z))
\end{aligned}
$$

where

$$
L_c(\gamma; y, z) = \sum_{i=1}^{n} z_{i0}\log\left(\frac{\pi_{i0}}{1-\pi_{i0}}\right) + \log\left(1-\pi_{i0}\right),
$$

$$
L_c(\beta_1, \theta_1; y, z) = \sum_{i=1}^{n} z_{i1}\left[\log(\pi_1) + \log\left(\frac{\Gamma(y+\theta_1)}{y_i!\Gamma(\theta_1)}\left(\frac{\theta_1}{\mu_{i1}+\theta_1}\right)^{\theta_1}\left(\frac{\mu_{i1}}{\mu_{i1}+\theta_1}\right)^{y_i}\right)\right],
$$

and

$$L_c(\beta_2, \theta_2; y, z)) = \sum_{i=1}^{n} z_{i2} \left[\log(\pi_2) + \log \left(\frac{\Gamma(y+\theta_2)}{y_i!\Gamma(\theta_2)} \left(\frac{\theta_2}{\mu_{i2}+\theta_2} \right)^{\theta_2} \left(\frac{\mu_{i2}}{\mu_{i2}+\theta_2} \right)^{y_i} \right) \right].$$

Substituting in the link functions for μ_{i1}, μ_{i2} and π_{i0}, we have

$$L_c(\gamma; y, X_0, z) = \sum_{i=1}^{n} z_{i0} X_{i0}\gamma - \log\left(1 + e^{X_{i0}\gamma}\right),$$

$$L_c(\beta_1, \theta_1; y, z, X_1) = \sum_{i=1}^{n} z_{i1} \left[\log(\pi_1) + \log \left(\frac{\Gamma(y+\theta_1)}{y_i!\Gamma(\theta_1)} \left(\frac{\theta_1}{e^{X_{i1}\beta_1}+\theta_1} \right)^{\theta_1} \left(\frac{e^{X_{i1}\beta_1}}{e^{X_{i1}\beta_1}+\theta_1} \right)^{y_i} \right) \right],$$

and

$$L_c(\beta_2, \theta_2; y, X_2, z)) = \sum_{i=1}^{n} z_{i2} \left[\log(\pi_2) + \log \left(\frac{\Gamma(y+\theta_2)}{y_i!\Gamma(\theta_2)} \left(\frac{\theta_2}{e^{X_{i2}\beta_2}+\theta_2} \right)^{\theta_2} \left(\frac{e^{X_{i2}\beta_2}}{e^{X_{i2}\beta_2}+\theta_2} \right)^{y_i} \right) \right].$$

It is easy to see that we can separate out the complete log likelihood with respect to each component and their set of regression parameters. Thus, we can seek maximize each likelihood separately in the M-step (Lambert 1992, McLachan 1997).

1.3 E-Step

The Q-function for the E-step at iteration k is given as the expectation of the complete likelihood with respect to z_i, given the estimates of the model parameters from the M-step. This expectation is τ_{ij}^k, the posterior probability of component membership for component j, $j = 0, 1, 2$, at iteration k.

$$\begin{aligned} Q(\Psi; \Psi^{(k)}) &= \sum_{i=1}^{n} \tau_{i0}^{(k)}(y_i, \Psi^{(k)}) \log(\pi_{i0}) + \log(1-\pi_{i0}) \\ &+ \tau_{i1}^{(k)}(y_i, \Psi^{(k)}) \left[\log(\pi_1) + \log(f_1(y_i, \mu_{i1}(\Psi), \theta_1)\right] \\ &+ \tau_{i2}^{(k)}(y_i, \Psi^{(k)}) \left[\log(\pi_2) + \log(f_2(y_i, \mu_{i2}(\Psi), \theta_2)\right] \end{aligned}$$

$$E[z_{i0}|y_i, \Psi^{(k)}] = \tau_{i0}^{(k)}(y_i, \Psi^{(k)}) = \frac{\pi_{i0}^{(k)} f_i(y_i)}{T_i},$$

$$E[z_{i1}|y_i, \Psi^{(k)}] = \tau_{i1}^{(k)}(y_i, \Psi^{(k)}) = \frac{(1-\pi_{i0}^{(k)})\pi_1^{(k)} f_1\left(y_i, \mu_{i1}\left(\Psi^{(k)}\right), \theta_1^{(k)}\right)}{T_i},$$

$$E[z_{i2}|y_i, \Psi^{(k)}] = \tau_{i2}^{(k)}(y_i, \Psi^{(k)}) = \frac{(1-\pi_{i0}^{(k)})\pi_2^{(k)} f_2\left(y_i, \mu_{i2}\left(\Psi^{(k)}\right), \theta_2^{(k)}\right)}{T_i},$$

where

$$T_i = \pi_{i0} f_0(y_i) + (1-\pi_{i0}) \left[\pi_1^{(k)} f_1 \left(y_i, \mu_{i1} \left(\Psi^{(k)} \right), \theta_1^{(k)} \right) + \pi_2^{(k)} f_2 \left(y_i, \mu_{i2} \left(\Psi^{(k)} \right), \theta_2^{(k)} \right) \right].$$

Here,

$$f_0(y_i) = \begin{cases} 1 & y_i = 0 \\ 0 & y_i > 0 \end{cases}$$

pertains to whether the observed window read count y_i is zero,

$$\begin{aligned} f_1(y_i, \mu_{i1}\left(\Psi^{(k)}\right), \theta_1^{(k)}) &= \frac{\Gamma(y+\theta_1^{(k)})}{y_i!\Gamma(\theta_1^{(k)})} \left(\frac{\theta_1^{(k)}}{\mu_{i1}\left(\Psi^{(k)}\right) + \theta_1^{(k)}} \right)^{\theta_1^{(k)}} \left(\frac{\mu_{i1}\left(\Psi^{(k)}\right)}{\mu_{i1}\left(\Psi^{(k)}\right) + \theta_1^{(k)}} \right)^{y_i} \\ &= \frac{\Gamma(y+\theta_1^{(k)})}{y_i!\Gamma(\theta_1^{(k)})} \left(\frac{\theta_1^{(k)}}{e^{X_1\beta_1^{(k)}} + \theta_1^{(k)}} \right)^{\theta_1^{(k)}} \left(\frac{e^{X_1\beta_1^{(k)}}}{e^{X_1\beta_1^{(k)}} + \theta_1^{(k)}} \right)^{y_i}, \end{aligned}$$

pertains to the negative binomially distributed background component and

$$\begin{aligned} f_2(y_i, \mu_{i2}\left(\Psi^{(k)}\right), \theta_2^{(k)}) &= \frac{\Gamma(y+\theta_2^{(k)})}{y_i!\Gamma(\theta_2^{(k)})} \left(\frac{\theta_2^{(k)}}{\mu_{i2}\left(\Psi^{(k)}\right) + \theta_2^{(k)}} \right)^{\theta_2^{(k)}} \left(\frac{\mu_{i2}\left(\Psi^{(k)}\right)}{\mu_{i2}\left(\Psi^{(k)}\right) + \theta_2^{(k)}} \right)^{y_i} \\ &= \frac{\Gamma(y+\theta_2^{(k)})}{y_i!\Gamma(\theta_2^{(k)})} \left(\frac{\theta_2^{(k)}}{e^{X_2\beta_2^{(k)}} + \theta_2^{(k)}} \right)^{\theta_2^{(k)}} \left(\frac{e^{X_2\beta_2^{(k)}}}{e^{X_2\beta_2^{(k)}} + \theta_2^{(k)}} \right)^{y_i}, \end{aligned}$$

pertaining to the negative binomially distributed enrichment component. We can see that the posterior probabilities of component membership are adjusted for each window's set of covariates, their estimated effects in each component, and the estimated baseline effect of each component (intercept).

1.4 M-Step

Because the Q function with respect to each set of regression parameters is distinct, we can maximize each separately using weighted Generalized Linear Models. The weights for each component's regression model correspond to the calculated posterior probability of belonging to that component from the E-step. In this sense, a window's count is partitioned in relation to its component memebership vector $(\tau_{i0}, \tau_{i1}, \tau_{i2})$ and modeled by each component's regression model. This allows us to obtain component specific estimates of covariates from the same set of windows. We obtain the parameter model estimates in the manner as follows:

For $\gamma^{(k+1)}$: maximize

$$L_c(\gamma; y, z) = \sum_{i=1}^{n} \tau_{i0}^{(k)} X_{i0}\gamma - \sum_{i=1}^{n} \log\left(1 + e^{X_{i0}\gamma}\right)$$

Now, suppose n_0 of the y_i's are 0 such that $y_{i1}, ..., y_{in_0}$ are zero and $y_{i(n_0+1)}, ..., y_{in}$ are greater than zero. Then, specify a matrix $W^{(k)}$ with diagonal $w^{(k)} = (w_{n_0}^{(k)}, w_n^{(k)}) = (\tau_{i0}^{(k)}, ..., \tau_{n_0 0}^{(k)}, 1 - \tau_{(n_0+1)0}^{(k)}, ..., 1 - \tau_{n0}^{(k)})$, where $\tau_{i0}^{(k)}$ is the posterior probability of the ith observation belonging to the zero inflated component at iteration k. Then $\gamma^{(k+1)}$ can be calculated by weighted logistic regression for the response y for $y = 0$ vs. $y > 0$, where weight matrix $W^{(k)}$ reduces the maximization of the zero-inflated likelihood to weighted logistic regression (Lambert 1992).

For $\beta_1^{(k+1)}$: maximize

$$L_c(\beta_1, \theta_1; y, z) = \sum_{i=1}^{n} \tau_{i1}^{(k)} \left[\log(\pi_1) + \log \left(\frac{\Gamma(y + \theta_1)}{y!\Gamma(\theta_1)} \left(\frac{\theta_1}{e^{X_{i1}\beta_1} + \theta_1} \right)^{\theta_1} \left(\frac{e^{X_{i1}\beta_1}}{e^{X_{i1}\beta_1} + \theta_1} \right)^{y} \right) \right].$$

Then, $\beta_1^{(k+1)}$ can be calculated by running a weighted negative binomial regression for the response y with prior weights $\tau_1^{(k)}$ (Lambert 1992, McLachlan 2007). Weighted negative binomial regression maximizes the above likelihood also for $\theta_1^{(k+1)}$ similar to the iterative method described in Hilbe, 2007. Also, $\beta_2^{(k+1)}$ and $\theta_2^{(k+1)}$ are maximized in a similar fashion. Lastly,

$$\pi_{i0}^{(k+1)} = \frac{e^{X_{i0}\gamma^{(k)}}}{1 + e^{X_{i0}\gamma^{(k)}}},$$

$$\pi_1^{(k+1)*} = \frac{\sum_{i=1}^{n} \tau_{i1}}{(\sum_{i=1}^{n} \tau_{i1} + \tau_{i2})},$$

and

$$\pi_2^{(k+1)} = \frac{\sum_{i=1}^{n} \tau_{i2}}{\sum_{i=1}^{n} (\tau_{i1} + \tau_{i2})}$$

For identifiability reasons, we place a constraint on π_1 such that

$$\pi_1^{(k+1)} = \max \left(\pi_{1,min}, \pi_1^{(k+1)*} \right)$$

where $\pi_{1,min}$ is chosen to be 0.5.

1.5 Convergence

We cycle between calculating posterior probabilities of component membership in the E-step and estimation of component-specific effects in the M-step until model convergence. The model terminates until the relative change in the complete model log-likelihood at iteration k compared to $k - 10$ is less than 10^{-5}.

1.6 Robustness of Model Initialization

In some cases the EM algorithm may converge to a local optimum due to poor initialization of the model parameters. We demonstrate the robustness of our initialization procedure by applying various starting partitions to the K562

Table 1: Final ZINBA Background parameter estimates for several initial partitions

Initialization Prop. Of Enrichment	0.001	0.01	0.05	0.10	0.15
(Intercept)	-1.455	-1.455	-1.455	-1.455	-1.436
Mappability	1.356	1.356	1.356	1.355	1.347
Local BG Estimate	0.867	0.867	0.867	0.867	0.867
GC-content	-0.797	-0.798	-0.797	-0.797	-0.820
Mappability*Local BG Estimate	0.269	0.268	0.269	0.269	0.268

FAIRE-seq data from chromosome 22, and compare the resulting model estimates after convergence. Each partition selects a different proportion of the top-ranked windows in terms of read counts and assigns them to the enrichment component. All other windows with non-zero count are assigned to background, and all zero count windows are assigned to the zero inflated component. As described in the main text, this partitioning of the data into each component is used to initialize the subsequent component-specific parameter estimates. The final model parameter estimates are robust to initialization (Table 1) in each component. We show only the final parameter estimates from the background component, as the results are similar for the other components. The BIC-selected model selected for this dataset in the main text was utilized.

2 Calculation of Local Background Estimates in ZINBA

The goal of the local background estimate is to approximate fluctuations in non-enriched regions from *-seq data, including changes related to copy number variations (CNV). Here large (100 kb by default) windows are tiled across each chromosome at a user defined step size (2.5 kb, default). For each large window the mappability-adjusted count of reads is calculated such that $\frac{n_{reads}}{n_{mappable}}$, where n_{reads} is the number of reads falling into a large window and $n_{mappable}$ are the number of bases in the window that are mappable by the users mappability criteria. The sliding window approach provides a good smoothed approximation of changes in background levels. For each smaller window used, the local background estimate is simply the average mappability-adjusted count for all overlapping large window times the length of smaller window.

However, the change in signal at the boundaries of CNVs is abrupt and the resulting smoothed background

estimate for ZINBA windows in the surrounding regions is artificially inflated outside of the CNV and deflated inside the CNV. This is because large windows that overlap this boundary straddle amplified and non-amplified background regions, providing a less accurate estimate of local background in the region just outside the CNV and just inside the CNV. To compensate for these boundary effects we attempt to identify CNV boundaries based on the resulting increase in variance of the local background estimates for ZINBA windows typically observed near CNVs. We compare the chromosome-wide global variance for all overlapping large windows in a local region. For each comparison of local versus global variance we use a statistic

$$F_i = \frac{\sigma^2_{global}/(n_{global} - 1)}{\sigma^2_{local,i}/(n_{local,i} - 1)}$$

where i is the index of the local region, n_{global} is the number of large windows and $n_{local,i}$ is the number of overlapping large windows in given location i. We choose threshold corresponding to a 95 percentile of an F(n_{global},n_{local}) distribution.

The exact CNV boundary is then determined using two contiguous windows slid across the candidate region, where a binomial test is employed to assess the equality of counts between windows. The position with the lowest p-value is called as the boundary, as this position would have the largest difference in count between the contiguous windows. This is because one window would reside completely in a normal background region and the other would reside completely within the CNV region. All overlapping large windows are removed and a refined local background estimate is calculated for the surrounding regions originating away from the boundary.

3 Data Access and Details

All downloaded datasets are freely available and consisted of mapped reads corresponding to human genome build HG18. The CTCF ChIP-seq data was derived from the GM12878 cell line and consisted of 14 million experimental reads and 16 million input reads and was generated by the University of Texas-Austin, version 2. The RNA polymerase II ChIP-seq dataset was derived from the K562 cell line and consisted of 22 million experimental reads and 16 million input reads and was also generated by the University of Texas-Austin, version 2. Reads mapping only uniquely to the genome were kept and an average fragment library length of 200 base pairs was assumed for ZINBA. The FAIRE-seq data was also from the K562 cell line and consisted of 52 million experimental reads and no input reads were available. Reads mapping up to four locations in the genome were kept and an average fragment length of 134 base pairs was used. Links for data downloads are provided below:

- UT-Austin GM12878 CTCF ChIP-seq `http://hgdownload.cse.ucsc.edu/goldenPath/hg18/encodeDCC/wgEncodeChromatinMap/wgEncodeUtaChIPseqAlignmentsRep3K562CtcfV2.tagAlign.gz`

- UT-Austin K562 RNA Pol II ChIP-seq `http://hgdownload.cse.ucsc.edu/goldenPath/hg18/encodeDCC/wgEncodeChromatinMap/wgEncodeUtaChIPseqAlignmentsRep2K562Pol2.tagAlign.gz`

- UT-Austin GM12878 CTCF ChIP-seq Input Control `http://hgdownload.cse.ucsc.edu/goldenPath/hg18/encodeDCC/wgEncodeChromatinMap/wgEncodeUtaustinChIPseqAlignmentsK562Input.tagAlign.gz`

- UNC K562 FAIRE-seq `http://hgdownload.cse.ucsc.edu/goldenPath/hg18/encodeDCC/wgEncodeChromatinMap/wgEncodeUncFAIREseqAlignmentsRep2K562V2.tagAlign.gz`

MCF-7 FAIRE-seq data is not yet available on ENCODE but can be downloaded from `http://code.google.com/p/zinba/downloads/list`, in addition to the Duke K562 DNase Hypersensitivity sites.. Only MCF-7 FAIRE-seq data pertaining to chromosome 20 was used in this manuscript, so we make data corresponding to this chromosome available.

The histone H3 lysine 36 tri-methylation (H3K36me3) ChIP-seq dataset was obtained from combining two replicates from the K562 cell line resulting in 30 million experimental reads, version 2. Input controls for replicates were similarly combined, resulting in 22 million reads. Reads mapping up to 10 places in the genome were kept and an average fragment library length of 200 base pairs was used for ZINBA. This data was taken from the Broad Institute ENCODE group, and data downloads are available below:

- Broad Institute K562 H3K36me3 ChIP-seq Replicate 1 `http://hgdownload.cse.ucsc.edu/goldenPath/hg18/encodeDCC/wgEncodeBroadChipSeq/wgEncodeBroadChipSeqAlignmentsRep1K562H3k36me3V2.tagAlign.gz`

- Broad Institute K562 H3K36me3 ChIP-seq Replicate 2 `http://hgdownload.cse.ucsc.edu/goldenPath/hg18/encodeDCC/wgEncodeBroadChipSeq/wgEncodeBroadChipSeqAlignmentsRep2K562H3k36me3V2.tagAlign.gz`

- Broad Institute K562 Input Control Replicate 1 `http://hgdownload.cse.ucsc.edu/goldenPath/hg18/encodeDCC/wgEncodeBroadChipSeq/wgEncodeBroadChipSeqAlignmentsRep1K562ControlV2.tagAlign.gz`

- Broad Institute K562 Input Control Replicate 2 `http://hgdownload.cse.ucsc.edu/goldenPath/hg18/encodeDCC/wgEncodeBroadChipSeq/wgEncodeBroadChipSeqAlignmentsRep2K562ControlV2.tagAlign.gz`

RNA-seq data for the K562 cell line was derived by the Caltech ENCODE group, where the 75-nt paired end Raw RNA-seq isoform levels in reads per kilobase per million reads (RPKM) format was used. Downloads can be found at `http://hgdownload.cse.ucsc.edu/goldenPath/hg18/encodeDCC/wgEncodeCaltechRnaSeq/wgEncodeCaltechRnaSeqRawSig`[illegible] `wig.gz`.

CNV regions from K562 cells were also obtained from `http://hgdownload.cse.ucsc.edu/goldenPath/hg18/encodeDCC/wgEncodeHudsonalphaCnv/wgEncodeHudsonalphaCnvRegionsK562V2.bed.gz`

4 References

McLachlan, G. On the EM algorithm for overdispersed count data. Stat Methods Med Res 1997; 6; 76

Lambert, D. Zero-inflated Poisson regression, with an application to defects in manufacturing. Technometrics, FEBRUARY 1992, VOL. 34, NO. 1

Hilbe, Joseph M., Negative Binomial Regression, Cambridge, UK: Cambridge University Press (2007)
