# Supplementary material for: ZINBA integrates local covariates with DNA-seq data to identify broad and narrow regions of enrichment, even within amplified genomic regions
Source: Genome Biol. 2011 Jul 25;12(7):R67. doi: 10.1186/gb-2011-12-7-r67 (PMC3218829; doi:10.1186/gb-2011-12-7-r67)
Supplement: Additional file 3 — ZINBA version 2.0. A freeze of the ZINBA software as of 9 July 2011, which is included in the manuscript for archival purposes only. We recommend that users download ZINBA from our website [46] to ensure that the most up-to-date version is installed. [file gb-2011-12-7-r67-S3.GZ › zinba/src/maf.doc]

Specification of the Multiple Alignment Format (.maf) v1

Introduction

The multiple alignment format stores a series of multiple

alignments in a format that is easy to parse and relatively

easy to read. We apologize for creating yet another

multiple alignment format. We realize that there are

many in existence. What motivates this format is

to store multiple alignments at the dna level between

entire genomes. The existing formats we are aware of

are geared towards multiple alignments of single proteins,

and would require considerable extension at the least to

cope with genomic issues such as forward and reverse

strand directions, multiple pieces to the alignment,

and so forth.

A Simple Example

Here is a simple example of a file with two alignments

with three sequences each:

##maf version=1 scoring=probability

#mblastz 8.91 02-Jan-2005

a score=0.128

s human_hoxa 100 9 + 100257 ACA-TTACTA

s horse_hoxa 120 10 - 98892 ACAATTGCTA

s fugu_hoxa	88 8 + 90788 ACA--TGCTA

a score=0.071

s human_unc 9077 8 + 10998 ACAGTATT

s horse_unc 4555 6 - 5099 ACA--ATT

s fugu_unc 4000 4 + 4038 AC----TT

General Structure

The .maf format is line oriented. Multiple alignments each

end with a blank line. Each sequence in an alignment

is on a single line, which can get quite long. Words in

a line are delimited by any white space. Lines

starting with # are considered comments. Lines starting

with ## can be ignored by most programs, but contain

meta-data of one form or another.

The file is broken into paragraphs that terminate in a

blank line. Within a paragraph the first word of a line

indicates it's type. Each multiple alignment is in

a paragraph by itself that begins with an 'a' line and

which contains an 's' line for each sequence in the

multiple alignment. Parsers for now should ignore

other types of paragraphs, and other types of lines within

an alignment paragraph. Parsers should also ignore extra

blank lines before paragraphs.

The First Line

The first line of a .maf file begins with ##maf. This

word is followed by whitespace separated variable=value pairs.

There must *not* be white space surrounding the =.

The currently defined variables are:

version - Required. Currently set to one.

scoring - Optional. A name for the scoring scheme used for the

alignments. The current scoring schemes are:

bit - roughly corresponds to blast bit values - roughly

2 points per aligning base minus penalties for mismatches

and inserts.

blastz - blastz scoring scheme - roughly 100 points per aligning

base.

probability - some score normalized between 0 and 1.

program - Optional. Name of program generating alignment.

Parsers ignore variables they do not understand.

'a' Lines

Each alignment begins with an 'a' line. The 'a' is followed

by name=value pairs. There are no required name=value pairs.

The currently defined variables are:

score - Optional. Floating point score. If this is present

it is good practice to also define scoring in the first

line.

pass - Optional. Positive integer value. For programs that

do multiple pass alignments such as blastz,

this shows which pass this alignment came from.

Typically the pass 1 will find the strongest alignments

genome-wide, and pass 2 will find weaker alignments

between two first pass alignments.

's' Lines

The s lines together with the a lines define a multiple alignment.

The s lines have the following fields which are defined by

position rather than name=value pairs.

src - The name of one of the source sequences for the alignment.

Typically this will be the first word following the '>' in

a fasta file that was input to the multiple aligner.

start - The start of the aligning region in the source sequence.

This is a zero based number. If the strand field below is

'-' then this is the start relative to the reverse-complemented

source sequence.

size - The size of the aligning region in the source sequence. This

number is equal to the number of non-dash characters in the

alignment text field below.

strand - Either '+' or '-'. If '-' then the alignment is to the

reverse complemented source.

srcSize - The size of the entire source sequence, not just the parts

involved in the alignment.

text - The bases (or amino acids) in the alignment and any dashes

as well.

'i' Lines

The i lines are optional. They are used by the browser to display synteny

breaks. An i line must immediately follow the s line that it describes

(unless there is no s line for that species in the current block).

The i lines have the following fields which are defined by

position rather than name=value pairs.

src - The name of one of the source sequences for the alignment.

This should match the s line above the i line.

leftStatus - the syntenic status of the alignment before us vis a vis ourselves.

Possible Values:

MAF_INVERSE_STATUS	'V'

MAF_INSERT_STATUS	'I'

MAF_DUP_STATUS	'D'

MAF_NEW_STATUS	'N'

MAF_NEW_NESTED_STATUS	'n'

MAF_CONTIG_STATUS	'C'

MAF_TANDEM_STATUS	'T'

leftLen - length related information for the previous alignment for the species

rightStatus - the syntenic status of the alignment after us vis a vis ourselves

Possible values are same as leftStatus.

rightLen - length related information for the following alignment for the species

'q' Lines

The q lines are optional. They are used to display quality data for a

sequence. Like the i lines, q lines follow the s line that it describes.

The q lines contain the following fields:

src - The name of one of the source sequences for the alignment.

This should match the s line above the q line.

quality - The quality data for corresponding to the sequence on the s line.

As we are generally interested in bases with low quality, the quality

data in the maf is a compressed version of the actual quality data.

The quality data in the maf is:

min( floor(actualy quality value/5), 9)

This allows us to show more of the low-quality values. The relationship

between quality characters in the maf and the actualy quality value are

summarized in the following table:

.: In Gap Q == FAKE_GAP_QUAL

0: 0 <= Q < 5 || Q == 98

1: 5 <= Q < 10

2: 10 <= Q < 15

3: 15 <= Q < 20

4: 20 <= Q < 25

5: 25 <= Q < 30

6: 30 <= Q < 35

7: 35 <= Q < 40

8: 40 <= Q < 45

9: 45 <= Q < 98

F: Q == 99

'e' Lines

The 'e' lines are optional and indicate that there isn't aligning

DNA for a species but that the current block is bridged by a chain

that connects blocks before and after this block.

src -- The name of one of the source sequences for the alignment.

start -- The start of the non-aligning region in the source sequence.

This is a zero-based number. If the strand field is '-' then this is

the start relative to the reverse-complemented source sequence.

size -- The size in base pairs of the non-aligning region in the

source sequence.

strand -- Either '+' or '-'. If '-', then the alignment is to the

reverse-complemented source.

srcSize -- The size of the entire source sequence, not just the parts

involved in the alignment. alignment and any insertions (dashes)

as well.

status -- A character that specifies the relationship between the

non-aligning sequence in this block and the sequence that appears

in the previous and subsequent blocks.

The status character can be one of the following values:

C -- the sequence before and after is contiguous implying that this

region was either deleted in the source or inserted in the

reference sequence. The browser draws a single line or a '-'

in base mode in these blocks.

I -- there are non-aligning bases in the source species between

chained alignment blocks before and after this block. The

browser shows a double line or '=' in base mode.

M -- there are non-aligning bases in the source and more than 95%

of them are Ns in the source. The browser shows a pale yellow bar.

n -- there are non-aligning bases in the source and the next aligning

block starts in a new chromosome or scaffold that is bridged by

a chain between still other blocks. The browser shows either a

single line or a double line based on how many bases are in the

gap between the bridging alignments.

'r' Lines

The r lines are optional. They specify how this region of the

MAF was defined. A paragraph can only have one r line. The

first word defines the source type, with the remanded of the

words specific to the source type. The following source types

are defined:

txupstream - Gene transcription start upstream regions:

r txupstream size geneId

size - is the number of bases in the region

geneId - gene identifier for the gene
